# Activated MEK/ERK Pathway Drives Widespread and Coordinated Overexpression of UHRF1 and DNMT1 in Cancer cells

**DOI:** 10.1038/s41598-018-37258-3

**Published:** 2019-01-29

**Authors:** Jialun Li, Ruiping Wang, Xueli Hu, Yingying Gao, Zhen Wang, Jiwen Li, Jiemin Wong

**Affiliations:** Shanghai Key Laboratory of Regulatory Biology, Fengxian District Central Hospital-ECNU Joint Center of Translational Medicine, Institute of Biomedical Sciences and School of Life Sciences, East China Normal University, Shanghai, 200241 China

## Abstract

The UHRF1-DNMT1 axis plays a key role in DNA maintenance methylation in mammals. Accumulative studies demonstrate that UHRF1 is broadly overexpressed in cancers, which contributes to cancer cell proliferation and tumorigenesis. Interestingly, a proteasome-dependent downregulation of UHRF1 has been observed in pluripotent ground state mouse embryonic stem cells (mESCs) cultured in the presence of two kinase (MEK1/MEK2 and GSK3β) inhibitors (termed 2i), raising the question whether UHRF1 is similarly regulated in cancer cells. Here we present evidence that while addition of 2i broadly downregulates UHRF1 and DNMT1 in various cancer cells, distinct underlying mechanisms are involved. In contrast to mESCs, 2i-induced downregulation of UHRF1 and DNMT1 in cancer cells cannot be rescued by proteasome inhibitor and occurs primarily at the level of transcription. Furthermore, downregulation of UHRF1 and DNMT1 by 2i is due to inhibition of MEK1/MEK2, but not GSK3β activity. Data mining reveals a marked co-expression of UHRF1 and DNMT1 in normal tissues as well as cancers. We provide evidence that multiple transcription factors including E2F1 and SP1 mediate the transcriptional activation of UHRF1 and DNMT1 by the activated MEK/ERK pathway. Together our study reveals distinct regulation of UHRF1/DNMT1 in mESCs and cancer cells and identifies activated MEK/ERK pathway as a driving force for coordinated and aberrant over-expression of UHRF1 and DNMT1 in cancers.

## Introduction

Epigenetic changes are increasingly considered as valuable targets for cancer therapies^1^. DNA methylation, catalyzed by DNA methyltransferase enzymes (DNMTs), is one of the most consistent and best known epigenetic modifications in mammals^2^. Compared with normal cells, cancer cells often have global DNA hypomethylation and regional hypermethylation^3^. Although the exact mechanisms remain elusive, DNA methylation abnormalities in cancer cells are intimately linked to aberrant expression and function of DNA methylation machinery. In mammalian cells DNA methylation is maintained by coordinated functions of DNMT1, DNMT3A and DNMT3B, among them DNMT1 plays a dominant role in genome-wide DNA methylation maintenance^4^. The maintenance methylation by DNMT1 requires an accessory factor UHRF1, also known as ICBP90 in human and NP95 in mouse, which is essential for targeting DNMT1 to DNA replication forks^5,6^.

Elevated expression of DNMTs, especially DNMT1, has been observed in various cancer tissues and cancer cell lines^4,7–9^. Multiple mechanisms, including inactivation of the pRB pathway, activation of E2F family transcription factors^10,11^ and desregulation of p53, SP1 and SP3^12,13^ can lead to elevated DNMT1 expression. In addition, down-regulation of regulatory microRNAs such as miR-148 and miR-152^14,15^ also contribute to aberrant DNMT1 overexpression. Like DNMT1, UHRF1 overexpression has also been found in various cancers and associated with down-regulation of several tumor suppressor genes (TSG) including RB1^16^, p16^INK4^^17,18^, BRCA1^19^, PPARG^20^ and KiSS1^21^. In fact, multiple studies have identified UHRF1 overexpression as a powerful marker for cancer diagnosis and prognosis^22^. Aberrant UHRF1 expression in cancer cells has been reported to be regulated transcriptionally by transcription factors such as E2F1^23,24^, E2F8^25^, SP1^26^ and FOXM1^27^, and post-transcriptionally by micro RNAs^28–33^. However, despite being functional in the same pathway and frequently overexpressed in cancers, it is not known if the expression of UHRF1 and DNMT1 is coordinately regulated and, if does, by what signaling pathway(s).

Mouse embryonic stem cells (mESCs) cultured with serum and leukemia inhibitory factor (LIF) or serum-free media supplemented with two small molecule inhibitors (2i) for GSK3β and MEK1/2 exhibit distinct pluripotency (primed vs naïve mESCs) and epigenetic patterns^34^. Numerous studies demonstrated that 2i mESCs is globally hypomethylated as compared to serum mESCs^35–38^. While active demethylation and impaired de novo DNA methylation have been previously implicated in the global demethylation during transition from primed to naïve mESCs in 2i medium, recent studies have identified impaired maintenance methylation, as a consequence of down-regulated UHRF1 protein, as the main cause^39,40^. In this regard, Ras/Raf/MEK/ERK signaling pathway is known to play a key role in transmission of proliferative signals from growth factors receptors or mitogens receptors. In many types of tumors, this signaling pathway is activated owing to mutations in KRAS, NRAS, and BRAF^41,42^. Activated ERK in turn phosphorylates many transcription factors and regulates their transcriptional activities^43^. The glycogen synthase kinase-3 (GSK-3), found initially associated with glycogen synthesis^44,45^, is a serine/threonine kinase that participates in regulation of diverse cellular activities. GSK-3 is overexpressed in various cancers including colorectal, hepatic, ovarian and pancreatic carcinoma^46^. The above findings in mESCs raise the question if MEK1/2 and/or GSK3β pathways regulate UHRF1 and consequently DNA methylation in cancer cells.

In this study, we have compared the effect of 2i on UHRF1 and DNMT1 expression in mESCs and human cancer cells. Unlike in mESCs, we found that 2i negatively regulates UHRF1 and DNMT1 at the level of transcription and does so through inhibition of MEK1/2. Furthermore, we provide evidence for widespread co-expression of UHRF1 and DNMT1 and activated MEK/ERK pathway as a driving force for frequent UHRF1/DNMT1 overexpression in cancers.

## Results

### 2i downregulates UHRF1 and DNMT1 in both mESCs and HCT116 cells but through distinct mechanisms

**Figure 1:**
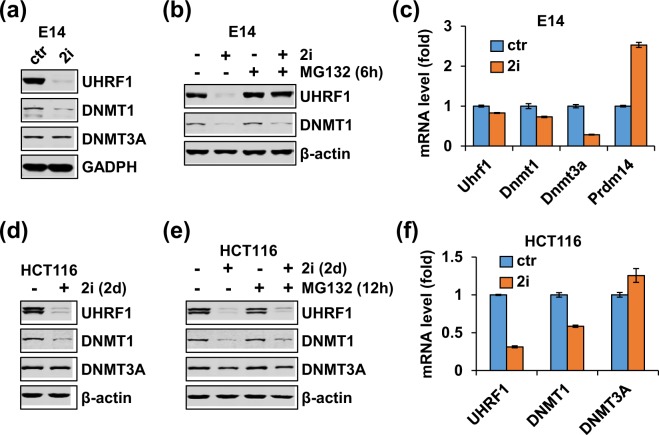
Comparison of 2i treatment on UHRF1 and DNMT1 expression in mESCs and HCT116 cells. (**a**) Western blotting analysis showing 2i down-regulated the levels of UHRF1 and DNMT1 proteins in mouse embryonic E14 cells. E14 cells were maintained in serum plus LIF with or without addition of 2i and subjected to western blotting analysis using antibodies as indicated. (**b**) Addition of MG132 blocked 2i-induced down-regulation of UHRF1. E14 cells maintained in serum plus LIF or 2i medium were treated with MG132 for 6 hours before harvested for western blotting analysis. (**c**) qRT-PCR analysis showing the 2i down-regulated the mRNA levels of DNMT3A, but not UHRF1 and DNMT1, in mouse embryonic E14 cells. E14 cells were maintained either in serum plus LIF or 2i medium and subjected to qRT-PCR analysis. (**d**) 2i also down-regulated UHRF1 and DNMT1 but not DNMT3A in HCT116 cells. The HCT116 cells were treated with or without addition of 2i for 2 days before harvested for western blotting analysis. (**e**) Addition of MG132 could not block 2i-induced down-regulation of UHRF1 and DNMT1 in HCT116 cells. The HCT116 cells were treated with or without addition of 2i for 2 days and MG132 was added 12 hours before cells were harvested. Western blotting analysis was performed using antibodies as indicated. (**f**) qRT-PCR analysis showing 2i down-regulated the mRNA levels of UHRF1 and DNMT1, but not DNMT3A, in HCT116 cells. The HCT116 cells treated with or without addition of 2i for 2 days were subjected to qRT-PCR analysis.

Previous studies have shown that the 2i-induced transition of “primed” mESCs to “naïve” mESCs is associated with a substantial reduction of UHRF1 protein^39,40^. We compared the levels of UHRF1, DNMT1 and DNMT3A proteins in mouse E14 ES cells that were cultured in serum plus LIF with or without addition of 2i (1 μM MEK1/2 inhibitor PD0325901 and 3 μM GSK3β inhibitor CHIR99021) by western blotting. We observed a marked reduction of UHRF1 and moderate reduction of DNMT1 in 2i culture condition (Fig. 1a), in agreement with previous reports^39,40^. Furthermore, addition of MG132, a proteasome inhibitor, 6 hours before cell harvest could fully restore the level of UHRF1 in 2i medium to the level of UHRF1 in serum plus LIF medium (Fig. 1b). On the other hand, MG132 treatment only partially restored the level of DNMT1. Quantitative RT-PCR analysis showed insignificant difference between the levels of Uhrf1 and Dnmt1 mRNAs under two different culture conditions (Fig. 1c). Interestingly, although the level of DNMT3A proteins remained constant, a reduced level of Dnmt3a mRNA was observed in 2i condition (Fig. 1c), possibly due to increased expression of PRDM14, which act as a DNMT3A transcriptional repressor^47^. Thus, our data are consistent with previous reports that 2i regulates UHRF1 primarily at the level of protein degradation and DNMT1 at both protein stability and transcription.

We next tested if 2i also regulates UHRF1 and DNMT1 in HCT116, a human colorectal carcinoma cell line. We found that, unlike E14 cells where addition of 2i did not affect cell proliferation, addition of 2i to culture medium inhibited HCT116 cell proliferation (Supplementary Fig. S1a) and increased the G0/G1 population (Supplementary Fig. S1b). We treated HCT116 cells with 2i for 2 days and examined the levels of UHRF1, DNMT1 and DNMT3A proteins by western blotting analysis. Similar to that in E14 cells, 2i treatment substantially decreased the levels of UHRF1 and DNMT1 proteins without affecting DNMT3A (Fig. 1d). However, addition of proteasome inhibitor MG132 could not rescue 2i-induced down-regulation of UHRF1 and DNMT1 proteins (Fig. 1e), suggesting that 2i negatively regulated UHRF1 and DNMT1 in HCT116 cells, but the regulation was not at the level of proteasome degradation. In support of this idea, quantitative RT-PCR analysis found that 2i treatment markedly reduced the mRNA levels of both UHRF1 and DNMT1, but not that of DNMT3A (Fig. 1f). Thus, while 2i-treatment down-regulated UHRF1 and DNMT1 primarily at the post-transcriptional level in mESCs, it negatively regulated UHRF1 and DNMT1 in HCT116 cells mainly at the level of transcription.

### 2i broadly regulates the transcription of both DNMT1 and UHRF1 in various cancer cells

**Figure 2:**
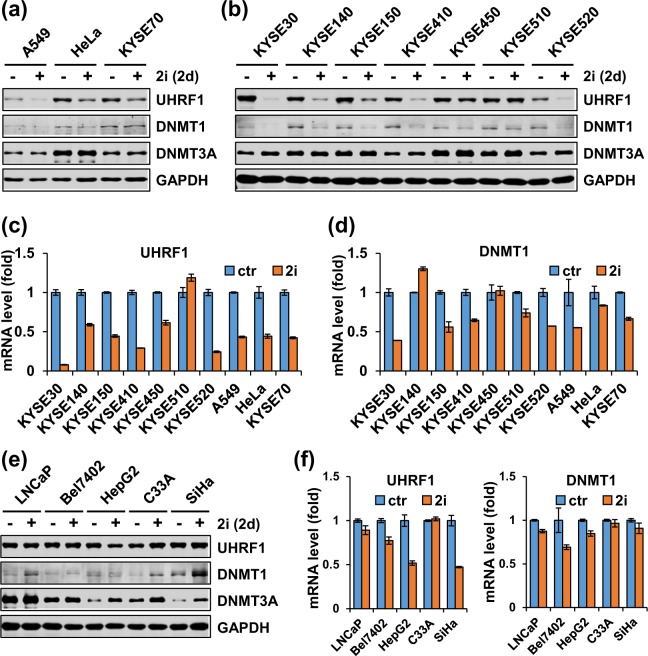
Addition of 2i broadly down-regulates UHRF1 and DNMT1 expression in various cancer cell lines. (**a**) Western blotting analysis showing addition of 2i down-regulated UHRF1 and DNMT1 proteins in A549, HeLa and KYSE70 cells. All the cells were cultured with or without addition of 2i for 2 days before harvested for western blotting analysis. (**b**) Western blotting analysis showing addition of 2i down-regulated UHRF1 and DNMT1 proteins in a panel of esophageal cells. (**c**,**d**) qRT-PCR analysis showing the effect of 2i on the levels of UHRF1 (**c**) or DNMT1 (**d**) mRNAs in various cancer cells. All the cells were cultured with or without addition of 2i for 2 days before harvested for qRT-PCR analysis. (**e**,**f**) Western blotting and qRT-PCR analysis showing the effect of 2i on the levels of UHRF1 and DNMT1 proteins (**e**) and mRNAs (**f**) in LNCaP, BEL7402, HepG2, C33A and SiHa cells. All the cells were cultured with or without addition of 2i for 2 days before harvested for western blotting and qRT-PCR analyses.

As distinct mechanisms were observed for regulation of UHRF1 and DNMT1 by 2i in mESCs and HCT116 cells, we wondered if and how 2i regulates UHRF1 and DNMT1 in various cancer cells. Furthermore, we surmised whether downregulation of UHRF1/DNMT1 by 2i could be explored for targeting aberrant DNA methylation in cancer cells. We thus treated A549 (lung cancer cell line), HeLa (cervical cancer cell line) and KYSE70 (esophageal cancer cell line) with 2i for 2 days and performed western blotting analysis for proteins and RT-PCR analysis for mRNA. Similarly, we carried out the same analyses using a panel of esophageal cancer cell lines. As shown in Fig. 2a, 2i treatment markedly down-regulated the levels of both UHRF1 and DNMT1 proteins in A549, HeLa and KYSE70 without affecting the level of DNMT3A proteins. Notably, 2i treatment also markedly down-regulated the levels of UHRF1 and DNMT1 proteins in all esophageal cancer cells (KYSE150, KYSE410, KYSE140, KYSE520, KYSE30, and KYSE450) with the only one exception of KYSE510, in which UHRF1 protein level was unchanged (Fig. 2b). Quantitative RT-PCR analysis revealed that 2i treatment induced a variable but significant reduction of UHRF1 transcripts in all above cancer cells with the exception of KYSE510 (Fig. 2c). Similarly, 2i treatment also induced a broad reduction of DNMT1 mRNA in most cancer cell lines, with exception of KYSE140 and KYSE450 (Fig. 2d). Also consistent with a less drastic reduction of DNMT1 vs UHRF1 in protein upon 2i treatment, 2i in general reduced DNMT1 mRNA less than that of UHRF1 (Fig. 2c,d). For DNMT3A, 2i treatment affected neither the levels of protein nor mRNA in majority of the cell lines, although a few of cancer cells showed increased levels of transcription (Supplementary Fig. S2). Together these data provide evidence that 2i broadly regulates UHRF1 and DNMT1 expression in various cancer cells, and does so primarily at the level of transcription. In support of this idea, we found that addition of MG132 could not rescued 2i-induced downregulation of UHRF1 and DNMT1 in A549, HeLa and KYSE70 cells (data not shown).

However, we found the regulation of UHRF1 and DNMT1 in cancer cells by 2i is not universal. Under the same experimental conditions, we found that 2i treatment did not cause a significant reduction of UHRF1 or DNMT1 proteins in LNCaP (prostate cancer), Bel7402, C33A and SiHa (cervical cancer) cells (Fig. 2e). Nevertheless, 2i treatment still caused a moderate to more severe reduction in transcription of UHRF1 in most of these cell lines (Fig. 2f), suggesting that additional mechanism(s) such as translation and protein stability may regulate the levels of UHRF1 proteins in these cell lines. Taken together, these data indicated that 2i can broadly but not universally suppress the transcription of DNMT1 and especially UHRF1 in various cancer cell lines.

### The MEK/ERK pathway, but not the GSK3β pathway, regulates UHRF1 and DNMT1 transcription in cancer cells

**Figure 3:**
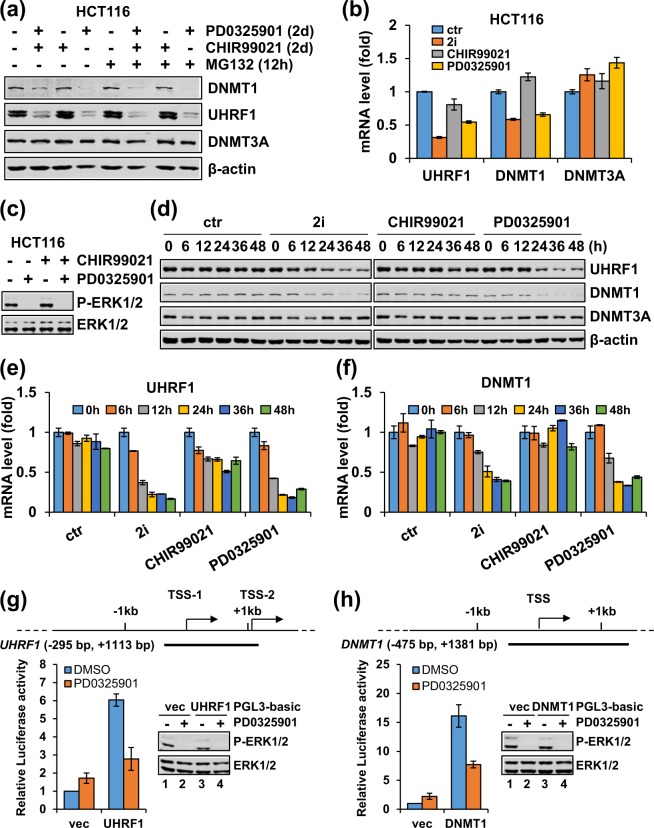
The MEK/ERK inhibitor PD0325901 is responsible for downregulation of UHRF1 and DNMT1 expression at the level of transcription in HCT116 cells. (**a**,**b**) HCT116 cells were treated without or with PD0325901 or CHIR99021 individually or in combination for 2 days before harvested for analysis of the levels of proteins (**a**) and mRNAs (**b**). (**c**) Western blotting analysis showing that PD0325901 treatment completely blocked ERK1/2 phosphorylation in HCT116 cells. The cells were treated with or without PD0325901 for 12 hours. (**d**–**f**) Time course experiments showing the kinetics of 2i, CHIR99021 and PD0325901 treatment on the down-regulation of UHRF1 and DNMT1 proteins (**d**), UHRF1 mRNA (**e**) and DNMT1 mRNA (**f**). Note that PD0325901 had the same effect as 2i. (**g**,**h**) Luciferase reporter assays showing the response of the UHRF1 and DNMT1 proximal promoters to MEK1/2 inhibitor PD0325901. The control PGL3-luc and PGL3-luc containing either UHRF1 (**g**) or DNMT1 (**h**) proximal promoter were transfected into HCT116 cells and treated with or without 1 μM PD0325901 for 24 hours before harvested for standard luciferase assay. The effect of PD0325901 on ERK1/2 phosphorylation was confirmed by western blotting analysis.

The 2i contains small molecular inhibitors CHIR99021 and PD0325901 that target GSK3β and MEK1/2, respectively. Having established that 2i broadly inhibits UHRF1 and DNMT1 expression in cancer cells, we next explored if GSK3β and/or MEK/ERK pathway were required for UHRF1/DNMT1 expression in cancer cells. We treated HCT116 cells separately or in combination with CHIR99021 and PD0325901 in the absence or presence of MG132. We found that similar to 2i treatment, PD0325901 alone reduced the levels of UHRF1 and DNMT1 proteins, and this reduction was not blocked by the proteasome inhibitor MG132 (Fig. 3a). In contrast, CHIR99021 treatment alone did not affect the protein levels of UHRF1 and DNMT1 (Fig. 3a). Furthermore, PD0325901 treatment caused an essentially equivalent reduction of UHRF1 and DNMT1 mRNAs as 2i, whereas CHIR99021 treatment alone had no effect (Fig. 3b). By western blotting analysis using an antibody reacting with Thr202/Tyr204 phosphorylated and active form of ERK1/2 (P-ERK1/2), the downstream target of MEK1/2, we confirmed PD0325901 and 2i effectively inhibited MEK1/2 activity (Fig. 3c). In contrast, neither 2i nor individual inhibitor significantly affected DNMT3A at the levels of both protein and transcription, suggesting that the MEK/ERK pathway specifically regulates the expression of UHRF1 and DNMT1 at the level of transcription.

To further characterize the regulation of UHRF1 and DNMT1 by MEK/ERK pathway, we performed time-course experiments. HCT116 cells were treated without or with 2i, PD0325901 and CHIR99021 for 0, 6, 12, 24, 36 and 48 hours, respectively. The subsequent western blotting analysis showed that both PD0325901 and 2i induced a time-dependent decrease of UHRF1 and DNMT1 proteins, with reduction of UHRF1 observed at 6 hours and peak at 36 hours and reduction of DNMT1 at 12 hours and peak around 36 hours (Fig. 3d). Again, CHIR99021 treatment did not affect the levels of UHRF1 and DNMT1 (Fig. 3d). Simultaneous RT-PCR analysis revealed that both PD0325901 and 2i induced a time-dependent progressive reduction of UHRF1 and DNMT1 mRNAs, with reduction of UHRF1 mRNA observed from 6 hours and DNMT1 from 12 hours (Fig. 3e,f). While CHIR99021 did not affect the level of DNMT1 mRNA throughout the experiments, it did moderately down-regulated the level of UHRF1 mRNA (Fig. 3e,f), although this effect on transcription did not appear to significantly affect the level of UHRF1 protein (Fig. 3d). Additional time-course experiments showed that PD0325901 markedly inhibited ERK1/2 phosphorylation ~ 1.5 hours (Supplementary Fig. S3a) after the addition of drug. We also found that MEK1/2 inhibitor PD0325901 did not affect the stability of UHRF1 and DNMT1 mRNAs (Supplementary Fig. S3b and S3c). Furthermore, although both 2i and PD0325901 inhibited cell proliferation and increased G0/G1 population of cells, we found that the levels of UHRF1 and DNMT1 mRNAs were not affected by cell cycle in HCT116 cells (Supplementary Fig. S3d and S3e). Given that reduced UHRF1 expression induced by both PD0325901 and 2i was observed within 6 hours treatment, we concluded that MEK/ERK pathway is most likely to directly regulate UHRF1 transcription, although we could not exclude the possibility that the MEK/ERK pathway could also regulate DNMT1 transcription indirectly.

To test that inhibition of MEK directly affects UHRF1 and DNMT1 transcription, we cloned the DNA fragments containing UHRF1 proximal promoter region (−295 bp to + 1113 bp) or DNMT1 proximal promoter region (−475 bp to + 1381 bp) into a promoter-less PGL3-basic luciferase reporter. Subsequent luciferase reporter assay in HCT116 cells showed that addition of PD0325901 inhibited transcriptional activity from the UHRF1 reporters (Fig. 3g) and the DNMT1 reporter (Fig. 3h), thus providing evidence for a direct transcriptional activation of UHRF1 and DNMT1 genes by the MEK/ERK pathway.

### The MEK/ERK pathway is generally required for elevated expression of UHRF1 and DNMT1 in cancer cells

**Figure 4:**
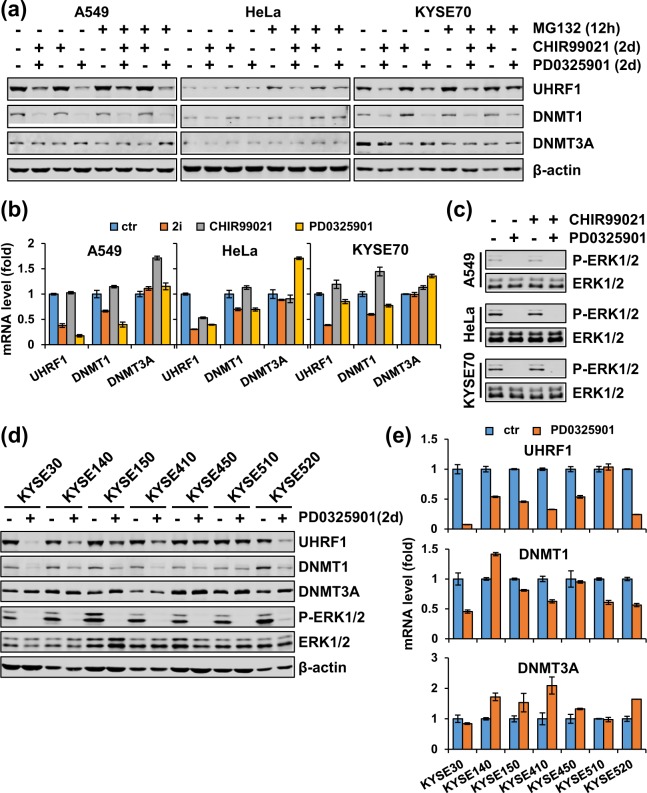
The MEK/ERK pathway broadly promotes UHRF1 and DNMT1 expression in cancer cells. (**a**) A549, HeLa and KYSE70 cells were first treated without or with PD0325901 or CHIR99021 individually or in combination for 36 hours and were then treated without or with MG132 for 12 hours before harvested for analysis of the levels of proteins by western blotting using antibodies as indicated. (**b**) A549, HeLa and KYSE70 cells were treated without or with PD0325901 or CHIR99021 individually or in combination for two days before cells were harvested for qPCR analysis for the levels of mRNAs for UHRF1, DNMT1 and DNMT3A. (**c**) Western blotting analysis showing that PD0325901 treatment completely blocked ERK1/2 phosphorylation in A549, HeLa and KYSE70 cells. The cells were treated with or without PD0325901 or CHIR99021 as indicated for 12 hours. (**d**,**e**) A panel of esophageal cancer cells were treated with PD0325901 for 2 days before harvested for analysis of the levels of various proteins by western blotting using antibodies as indicated (**d**) and the levels of mRNAs by qRT-PCR (**e**).

So far we have demonstrated that 2i has a broad effect on UHRF1 and DNMT1 expression in cancer cells and that the effect of 2i on UHRF1 and DNMT1 expression is due to specific inhibition of MEK/ERK pathway. We next attempted to determine if the MEK/ERK pathway is broadly required for elevated expression of UHRF1 and DNMT1 in cancer. To this end, we treated A549, HeLa and KYSE70 cells with CHIR99021, PD0325901 or both respectively. Subsequent western blotting analysis revealed that, like 2i, PD0325901 but not CHIR99021 was sufficient to induce down-regulation of UHRF1 and DNMT1, but not DNMT3A in all three cell lines (Fig. 4a). Addition of MG132 rescued neither 2i- nor PD0325901-induced down-regulation of UHRF1 and DNMT1 proteins (Fig. 4a). Furthermore, simultaneous RT-PCR analysis showed that PD0325901 inhibited UHRF1 and DNMT1 transcription to the similar extent as 2i (Fig. 4b). We also confirmed, as expected, that PD0325901 treatment drastically inhibited the level of P-ERK1/2 in these cells (Fig. 4c). Thus, the activated MEK/ERK pathway is required for elevated expression of UHRF1 and DNMT1 in these cancer cell lines.

To test the role of MEK/ERK pathway in transcriptional activation of UHRF1 and DNMT1 further, we also treated 12 different cancer cell lines with PD0325901 for 48 hours. We found that for 7 esophageal cancer cell lines PD0325901 treatment down-regulated both UHRF1 and DNMT1 at the level of proteins (Fig. 4d) and mRNAs (Fig. 4e) in all lines except KYSE510, a result that is essentially identical to that of 2i on UHRF1 and DNMT1 proteins (Fig. 2b) and mRNAs (Fig. 2c,d) in these cells. As expected, we found that PD0325901 treatment markedly inhibited the level of phosphorylated ERK1/2 in all these cells without affecting the level of ERK1/2 proteins (Fig. 4d). Similar to the results of 2i treatment in Fig. 2e, PD0325901 treatment failed to significantly affect the levels of UHRF1 and DNMT1 proteins in LNCaP, Bel7402, HeLa, C33A and SiHa cells (Supplementary Fig. S4a). Interestingly, the LNCaP cells showed a undetectable level of P-ERK1/2, which could explain the lack of effect of 2i and PD0325901 in this cell line (Supplementary Fig. S4a). Also similar to the results of 2i, PD0325901 treatment did reduce the level of UHRF1 and DNMT1 transcription in Bel7402 cells (Supplementary Fig. S4b). Taken together, these data provide compelling evidence that 2i broadly inhibits UHRF1 and DNMT1 expression in cancer cells by inhibition of the MEK/ERK pathway, which is broadly required for elevated transcription of both UHRF1 and DNMT1 in various cancer cells.

**Figure 5:**
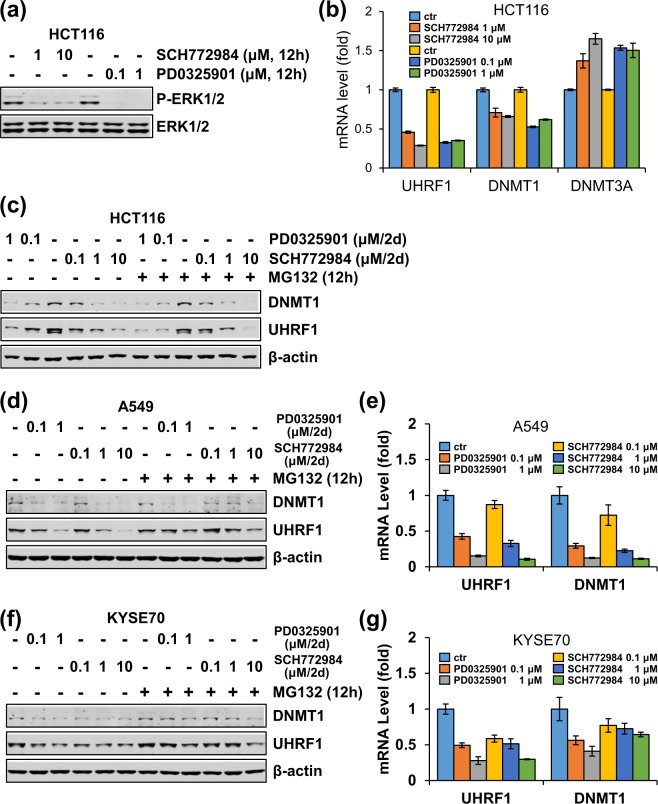
Like PD0325901, the MEK/ERK inhibitor SCH772984 can also down-regulate the expression of UHRF1 and DNMT1 at the level of transcription. (**a**,**b**) HCT116 cells were treated with different concentrations of PD0325901 or SCH772984 before cells were harvested for western blotting analysis for ERK1/2 and phosphorylated ERK1/2 (**a**), 12 hours) and the levels of mRNAs for UHRF1, DNMT1 and DNMT3A (**b**), 2 days). (**c**) HCT116 cells were first treated with different concentrations of PD0325901 or SCH772984 for 36 h and were then treated without or with MG132 for 12 hours before harvested for analysis of the levels of proteins by western blotting using antibodies as indicated. (**d**,**f**) A549 (**d**) and KYSE70 (**f**) cells were first treated with different concentrations of PD0325901 or SCH772984 for 36 hours and were then treated without or with MG132 for 12 hours before cells were harvested for western blotting using antibodies as indicated. (**e**,**g**) A549 (**e**) and KYSE70 (**g**) cells were treated with different concentrations of PD0325901 or SCH772984 for two days before cells were harvested for qPCR analysis for the levels of mRNAs for UHRF1, DNMT1 and DNMT3A.

To validate the specific effect of MEK/ERK pathway in regulation of UHRF1 and DNMT1, we made use of an ERK inhibitor SCH772984^48^. As expected, SCH772984 inhibited ERK1/2 activity in HCT116 cells in a dose-dependent manner (Fig. 5a). Like PD0325901, SCH772984 also induced down-regulation of UHRF1 and DNMT1 proteins and mRNAs (Fig. 5b,c) in HCT116 in a dose-dependent manner. Note that, like PD0325901, SCH772984-induced downregulation of UHRF1 and DNMT1 proteins was not rescued by MG132 (Fig. 5c). Similarly, we found that like PD0325901, SCH772984 also down-regulated UHRF1 and DNMT1 expression in A549 and KYSE70 cells (Fig. 5d–g). Thus, inhibition of either MEK1/2 or ERK1/2 down-regulated UHRF1 and DNMT1 expression in these cancer cells.

### UHRF1 and DNMT1 are coordinately expressed in various tissues and cellsh

**Figure 6:**
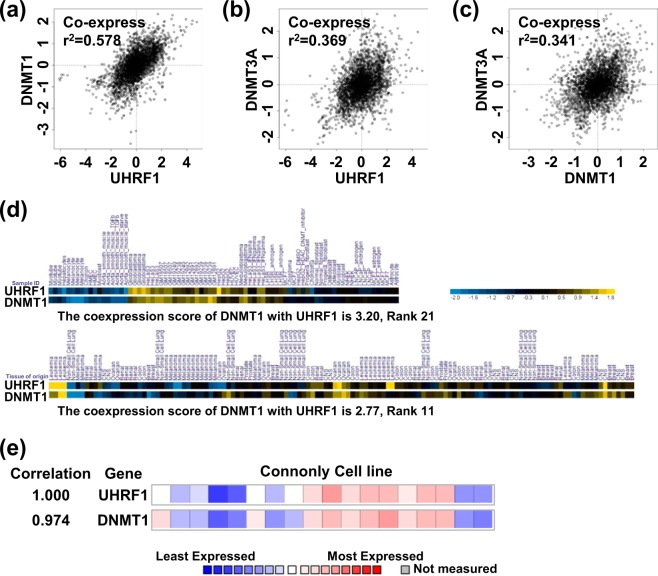
UHRF1 and DNMT1 are coordinately expressed in transcription. (**a**–**c**) The COXPRESdb was used for analyzing the co-expression status between UHRF1 and DNMT1 (**a**), DNMT3A and UHRF1 (**b**) and DNMT3A and DNMT1 (**c**). (**d**) The SEEK program was used for analyzing co-expression of UHRF1 and DNMT1 in dataset GSE15805 (upper panel) and dataset GSE29288 (lower panel). (**e**) Co-expression between UHRF1 and DNMT1 was analyzed with ONCOMINE database.

Our results this far showed that UHRF1 and DNMT1 are co-regulated at the level of transcription in various cancer cell lines via the MEK/ERK pathway. Given that both UHRF1 and DNMT1 are overexpressed in various cancers^8,9,22^, we hypothesized that UHRF1 and DNMT1, as the dual components of DNA maintenance methylation machinery, could be regulated coordinately at the level of transcription. The COXPRESdb (http://coxpresdb.jp) allows analysis of co-expression relationships for genes based on RNA-seq and microarray^49^. Using this program we analyzed the expression correlation between UHRF1 and DNMT1 in different cancer cell lines (brain, pituitary, and prostate) and normal tissues (bone marrow) in Homo sapiens. As shown in Fig. 6a, UHRF1 have high expression correlation with DNMT1 (r^2^ = 0.587). As a control, we also analyzed the co-expression between DNMT1 and DNMT3A, as a coordinated expression of DNMT1 and DNMT3A was reported previously^7^. However, compared to the high correlation of UHRF1 and DNMT1 expression, DNMT3A showed a much reduced correlation (r^2^ = 0.369) with UHRF1 (Fig. 6b), as well as with DNMT1 (r^2^ = 0.341) expression (Fig. 6c). A similar result was observed when co-expression among Uhrf1, Dnmt1 and Dnmt3a was analyzed in Mus musculus (Supplementary Fig. S5). Taken together, these data indicated that UHRF1 and DNMT1 are co-expressed in different tissues and cancers.

SEEK (search-based exploration of expression compendia; http://seek.princeton.edu/) also provides a means for analyzing gene co-expression^50^. We searched for the co-expressed genes for UHRF1 using this program and found that DNMT1 showed a high co-expression score with UHRF1 in cancer samples (3.20 in upper panel) and normal tissues (2.77 in lower panel) (Fig. 6d). Furthermore, a similar co-expression of UHRF1 and DNMT1 could be found in ONCOMINE database by analyzing the Connolly cell line and the correlation score is 0.974. (Fig. 6e). These data support the idea that UHRF1 and DNMT1 are to large extent co-regulated at the level of transcription.

### The downstream transcription factors for activation of UHRF1/DNMT1 expression by the MEK/ERK pathway

**Figure 7:**
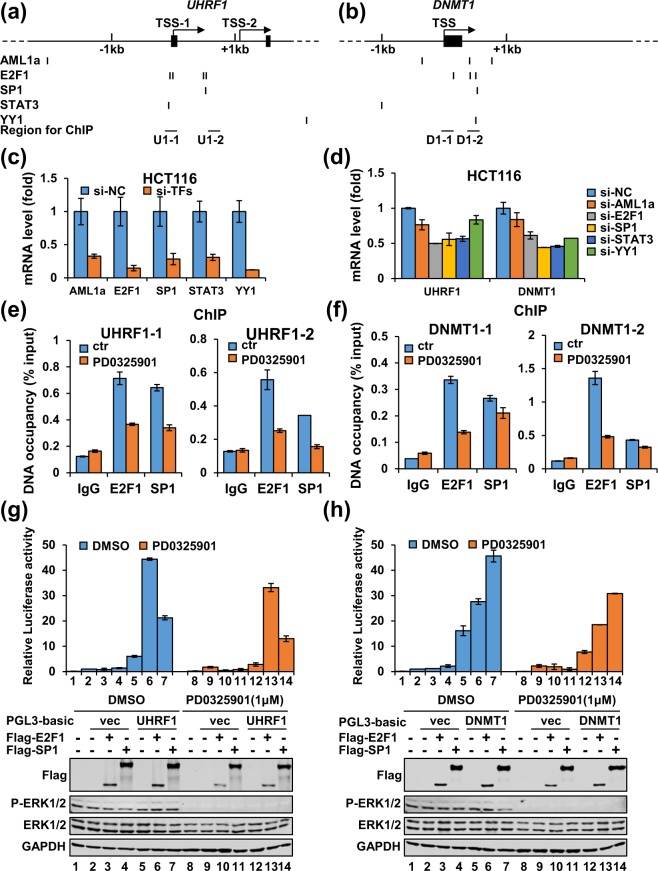
Multiple downstream transcription factors contribute to transcriptional activation of UHRF1 and DNMT1 by the MEK/ERK pathway. (**a**,**b**) The diagrams illustrating the putative binding sites for transcription factors AML1a, E2F1, SP1, STAT3 and YY1 within the region of −5kb and + 3 kb around the transcriptional start site of UHRF1 gene (**a**) and DNMT1 gene (**b**). (**c**) qRT-PCR analysis showing efficient knockdown of corresponding transcription factors in HCT116 cells by specific siRNA against AML1a, E2F1, SP1, STAT3 and YY1, respectively. (**d**) qRT-PCR analysis showing the effect on UHRF1 and DNMT1 expression in HCT116 cells upon knockdown of each of the transcription factors. (**e**,**f**) ChIP analysis showing the effect of PD0325901 treatment on the binding of E2F1 and SP1 to two regions within the UHRF1 promoter (**e**) or DNMT1 promoter (**f**). IgG, control immunoglobulin. (**g**,**h**). Luciferase reporter assay showing that E2F1 and SP1 markedly enhanced transcriptional activation from the UHRF1 and DNMT1 promoters. The control PGL3-luc and UHRF1-luc (**g**) or DNMT1-luc (**h**) were cotransfected with or without an E2F1 or SP1 expression plasmid into HCT116 cells and treated with or without PD0325901 as indicated. Also shown were western blotting data for corresponding samples.

Since UHRF1 and DNMT1 are co-expressed in different tissues and cancer cells and they are regulated at the transcription level by the MEK/ERK pathway, we hypothesized that they are co-regulated by a set of common transcription factors. To this end, we analyzed the transcription factor binding sites within −5 kb upstream and + 3 kb downstream of the transcription start site (TSS) of UHRF1 and DNMT1 genes using a software on QIAGEN website (http://www.sabiosciences.com/), which gives information on predicted binding sites for over 200 human transcription factors. This analysis revealed a series of common transcription factors shared by both UHRF1 and DNMT1 (Supplementary Fig. S6), among them 5 transcription factors AML1a^51^, E2F1^52^, SP1^53^, STAT3^54^ and YY1^55,56^ have been reported to be regulated by the MEK/ERK pathway (Fig. 7a,b). To test if these transcription factors indeed co-regulated both UHRF1 and DNMT1 transcription, we knocked down these transcription factors in HCT116 by corresponding specific siRNAs, and validated the knockdown efficiency by quantitative RT-PCR (Fig. 7c). We found that knockdown of each of the transcription factors led to reduced levels of UHRF1 and DNMT1 transcription, with more severe reduction of both UHRF1 and DNMT1 observed when E2F1, SP1 or STAT3 were knocked down (Fig. 7d). To test if these transcription factors also control UHRF1 and DNMT1 expression in other cancer cells, we carried out the same experiments in A549 cells. We found that knockdown of these transcription factors (Supplementary Fig. S7a) in general also resulted in variable reduction of UHRF1 and DNMT1 expression (Supplementary Fig. S7b). In multiple experiments we found that knockdown of single transcription factor such as E2F1 or SP1 did not lead to more than 50% reduction of UHRF1 or DNMT1, in supporting of the idea that the expression of UHRF1 and DNMT1 are likely regulated by a coordinated function of multiple transcription factors. Our attempts to simultaneously knockdown these transcription factors were not successful due to reduced knockdown efficiency for individual factors (data not shown). Thus, while our data indicate that the transcription of both UHRF1 and DNMT1 could be regulated by a common set of transcription factors including AML1a, E2F1, SP1, STAT3, and YY1, it remains to be determined how these factors coordinately regulate UHRF1 and DNMT1 expression.

It has been shown that the MEK/ERK pathway enhances downstream transcription factor activity by enhancing their binding to specific regulatory DNA elements of target genes through phosphorylation of the transcription factors^57–59^. We thus wished to address if the MEK/ERK pathway regulates transcription of both UHRF1 and DNMT1 through affecting the binding of MEK/ERK- regulated transcription factors to their promoter regions. As knockdown E2F1 and SP1 had a more severe effect on UHRF1 and DNMT1, we next focused on these two transcription factors to test if ERK1/2 indeed controls their ability to bind the regulatory regions of UHRF1 and DNMT1. According to the predicted E2F1 and SP1 binding sites within UHRF1 and DNMT1 genes, we designed chromatin immunoprecipitation-quantitative PCR (ChIP-qPCR) assay to test the binding of transcription factors E2F1 and SP1 to two regions at the UHRF1 and DNMT1 promoters (Fig. 7a). HCT116 cells were treated with or without MEK/ERK inhibitor PD0325901 and subsequent ChIP-qPCR analysis demonstrated a significant reduction of E2F1 and SP1 associated with both regions at the UHRF1 promoter (Fig. 7e). For DNMT1 gene, ChIP-qPCR results revealed a substantial reduction of binding of E2F1 but not SP1 (Fig. 7f). As knockdown of SP1 significantly down-regulated transcription of DNMT1 in HCT116 cells (Fig. 7d), we suggested that SP1 may regulate DNMT1 expression by binding to the site(s) other than the one we analyzed.

To test if E2F1 and SP1 directly activate UHRF1 and DNMT1 transcription, we carried out luciferase reporter assay. As shown in Fig. 7g,h, ectopic expression of E2F1 or SP1 markedly enhanced the luciferase activity from the UHRF1-luc reporter but not the control PGL3 reporter. Addition of PD0325901 inhibited transcriptional activation by E2F1 and SP1. Similar results were observed for the DNMT1-luc reporter. Thus, E2F1 and SP1 can activate UHRF1 and DNMT1 expression by binding to their proximal promoters and this activity is stimulated by the MEK/ERK pathway.

Taken together, we propose that the MEK/ERK pathway most likely controls UHRF1 and DNMT1 expression by enhancing the binding of downstream transcription factors such as E2F1 and SP1 to the regulatory regions of both UHRF1 and DNMT1.

## Discussion

Given the profound effect of 2i culture condition on mESCs pluripotency, DNA methylation and UHRF1/DNMT1 expression^35–40,47^, here we investigated if and how 2i regulates UHRF1/DNMT1 expression in cancer cells. We showed that 2i also regulates UHRF1/DNMT1 expression in cancer cells. But unlike the case in mESCs, 2i regulates UHRF1/DNMT1 expression in cancer cells primarily at the level of transcription. We further demonstrated that it is the MEK/ERK pathway that controls a coordinated transcription of UHRF1/DNMT1 in cancer cells.

While DNA hypomethylation phenotype in 2i cultured naïve mESCs were initially attributed to combinatorial effect of elevated expression of Tet family dioxygenases and impaired expression of de novo DNA methyltransferases^35–38,47^, recent studies identified impaired DNA maintenance, as a result of reduced UHRF1 protein stability, as the main cause of global demethylation in 2i cultured naive mESCs^39,40^. We found that 2i broadly down-regulated both UHRF1 and DNMT1, the axis of DNA maintenance methylation, but not de novo enzyme DNMT3A, in various cancer cells (Figs 1 and 2). However, we found that 2i down-regulated UHRF1 and DNMT1 expression primarily at the level of transcription (Figs 1 and 2). Furthermore, while 2i promotes UHRF1 proteasome degradation mediated by Pramel7 in mESCs^40^, we show here that the reduced UHRF1 and DNMT1 expression is due to impaired transcription and inhibition of the MEK/ERK pathway (Figs 3–5). In this regard, accumulative studies have demonstrated a widespread overexpression of UHRF1 in various cancer cells^22^, but the underlying mechanism is not well understood. As the MEK/ERK pathway is widely activated, as a result of RAS or BRAF mutations and others^60,61^, in human cancer cells, our finding provides a reasonable explanation for widespread transcriptional overexpression of UHRF1 (DNMT1 as well) in various cancers. In addition, this finding also provides an explanation for observed co-expression of UHRF1 and DNMT1 in our study. Notably, our co-expression analysis by database mining provides clear evidence for a widespread co-expression of UHRF1 and DNMT1 in normal tissues and numerous cancer cells (Fig. 6). In fact, by three different approaches, the co-expression score of UHRF1 and DNMT1 is higher than that of DNMT1 and DNMT3A, which was previously proposed to be coordinately co-expressed in order to maintain DNA methylation homeostasis in cancer cells^7^. As BRAF and RAS mutations, especially KRAS, broadly occur in cancers, it will be of interest to systematically analyze the relationship between UHRF1/DNMT1 expression and BRAF/RAS mutations. As the key essential components for DNA maintenance methylation, a coordinated expression of UHRF1 and DNMT1 would theoretically promote DNA methylation. However, although regional DNA hypermethylation can occur, cancer cells often exhibit a global DNA hypomethylation phenotype^3^. Thus, the regulation of DNA methylation homeostasis in cancers is likely complicated and influenced not only by the elevated expression of UHRF1/DNMT1 but also by other parameters such as the activity of UHRF1/DNMT1, expression and activity of de novo DNA methyltransferases and TET family proteins.

While long term 2i treatment induces global hypomethylation in mESCs^35–38^, we have not observed significant reduction of DNA methylation in cancer cells upon 2 day of 2i or longer treatment (data not shown). A pitfall in our study is that, while 2i treatment did not affect mESCs proliferation, it suppressed cancer cell proliferation (Supplementary Fig. S1 and data not shown). Similarly, we found that MEK1/2 inhibitor alone broadly suppressed cancer cell proliferation. This observation is consistent with numerous previous studies showing a key role for the activated RAS/RAF/MEK/ERK pathway in promoting cancer cell growth and proliferation^42^. This inhibitory effect on cancer cell proliferation could explain why we failed to detect a marked reduction of DNA methylation in 2i or PD0325901-treated cells (data not shown), as multiple rounds of DNA replication is likely required to observe accumulative effect of impaired DNA maintenance methylation. Alternatively, it is also possible that although 2i or PD0325901 treatment markedly down-regulated the levels of UHRF1 and DNMT1, it had yet to reach a low threshold level of UHRF1 and/or DNMT1 that is sufficient for maintaining DNA methylation in cells^4^.

We also provide evidence that multiple transcription factors are likely to mediate the transcriptional activation of UHRF1/DNMT1 by the MEK/ERK pathway and among the transcription factors are E2F1 and SP1. E2F1 and SP1 are regulated by the MEK/ERK pathway and have also been independently shown to regulate UHRF1 expression in previous studies^10,12,24,26,62^. We demonstrated that knockdown of E2F1 or SP1 down-regulated UHRF1 and DNMT1 expression and that treatment with ERK inhibitor PD0325901 impaired E2F1 and SP1 binding to the UHRF1 and DNMT1 promoters (Fig. 7 and Supplementary Fig. S7). It is noteworthy that throughout the study we generally observed a more severe effect of 2i or PD0325901 on expression of UHRF1 than DNMT1. Similarly, our ChIP-qPCR analysis also revealed a more severe effect of PD0325901 on binding of E2F1 and SP1 to the promoter of UHRF1. These observations are likely functionally relevant, as UHRF1 in general is more frequently and highly overexpressed in cancers than DNMT1. Furthermore, as the levels of DNMT1 in general is not a limiting factor for DNA methylation^4^, it is formally possible that UHRF1 rather than DNMT1 is the hub for regulation of DNA maintenance methylation.

## Methods

### Reagents and Antibodies

The reagents used in this study were as follows: MEK inhibitor PD0325901 (S1036) and GSK-3 inhibitor CHIR99021 (S2924) were purchased from Selleckchem (Houston, TX), ERK1/2 inhibitor SCH772984 (HY-50846) from MCE (Monmouth Junction, NJ), MG132 (M8699) from Sigma (St. Louis, MO) and Actinomycin D (HY-17559) from MCE(Monmouth Junction, NJ). The antibodies used in this study were as follows: Erk1/2 (4695), P-ERK1/2 (4370), E2F1 (3742) and SP1 (9389) from Cell Signaling Technology (Danvers, MA); DNMT1 (sc-20701) and DNMT3A (sc-20703) from Santa Cruz Biotechnology (Dallas, TX); UHRF1 (21402-1-AP) from Proteintech (Rosemont, IL); β-actin (M1210-2) from HUABIO (Cambridge, MA) and GAPDH (3B3) from Abmart (Berkeley Heights, NJ).

### Cell Culture and Inhibitors Treatment

A549, LNCaP, KYSE30, KYSE70, KYSE140, KYSE150, KYSE410, KYSE450, KYSE510 and KYSE520 cells were cultured in 1640 medium (Gibco). HeLa, C33A, BEL7402, HepG2 and HCT116 cells were cultured in DMEM medium (Gibco). The medium was supplemented with 10% fetal bovine serum (FBS, Gemini) and 1% penicillin/streptomycin (Invitrogen). E14 cells were cultured in DMEM supplemented with 15% fetal bovine serum (Gibco), 1000 U/mL leukaemia inhibitory factor (Millipore), 1% penicillin/streptomycin (Invitrogen), 1 × non-essential amino acids (Gibco), 1 mM sodium pyruvate (Gibco), 2 mM L-glutamine (Gibco) and 55 μM beta-mercaptoethanol (Sigma). The cultured cells were maintained at 37 °C in a humidified incubator with 5% CO_2_. For inhibitors treatment, PD0325901 was in general used at a final concentration of 1 μM and CHIR99021 was 3 μM otherwise as indicated. For mRNA half-life measurement experiment, the cells were treated with Actinomycin D at a final concentration of 5 μg/mL. DMSO treatment was as a negative control.

### Introduction of siRNAs into Cells

To knock down the indicated genes, siRNAs were transfected into cells twice within 24 hours using Lipofectamine 2000 (Invitrogen) according to the manufacturer’s instructions. The siRNA oligonucleotides were synthesized by GenePharma (Shanghai, China). siRNA sequences are listed below. 5′-AAGACAUCGGCAGAAACUA-3′ for human AML1a; 5′-GCUAUGAGACCUCACUGAAUC-3′ for human E2F1; 5′-GGAUGGUUCUGGUCAAAUACA-3′ for human SP1; 5′-CAACAUGUCAUUUGCUGAA-3′ for human STAT3; 5′-GGCAGAAUUUGCUAGAAUG-3′ for human YY1. The negative control (NC) siRNA sequence is 5′-UUCUCCGAACGUGUCACGUTT-3′.

### Western Blotting Analysis

The cells were directed lysed by 1 × SDS buffer (62.5 mM Tris-HCl, pH 6.8; 2% w/v SDS; 10% glycerol; 1% v/v beta-mercaptoethanol; 0.01% w/v bromophenol blue). Lysates were boiled at 95 °C for 15 min and then subjected to separate on 8% SDS-PAGE. Proteins were transferred to the nitrocellulose membrane (GE Healthcare Life Science), and the membranes were blocked with 8% milk for 1 hour at room temperature. After overnight incubation at 4 °C with primary antibodies (ERK1/2 (dilution: 1:1000); P-ERK1/2 (dilution: 1:1000); DNMT1 (dilution: 1:2000); DNMT3A (dilution: 1:3000); UHRF1 (dilution: 1:500);β-actin (dilution: 1:10000); GAPDH (dilution: 1:10000)), the membranes were washed three times with PBST buffer (0.1% Tween-20 in 1 × PBS), followed by incubation with Alexa Fluor® 680 goat anti-rabbit or Alexa Fluor® 790 goat anti-mouse antibody (Jackson ImmunoResearch, dilution: 1:10000) for 1 hour at room temperature. The membranes were visualized by the Odyssey CLx Imaging System (LI-COR Bioscience).

### Chromatin Immunoprecipitation and Quantitative PCR (ChIP-qPCR)

The HCT116 cells with or without inhibitor treatment were crosslinked with 1% formaldehyde for 15 min before neutralization with 0.125 M glycine. The cells were lysed by sonication in ChIP Lysis buffer (25 mM Tris-HCl pH 8.0, 0.1% SDS, 1 mM EDTA, 1 × protease inhibitor cocktail (MCE)), and soluble cell extracts were recovered after centrifugation at 12000 rpm and 4 °C for 20 min. The indicated antibodies (E2F1 or SP1) were added, followed by overnight incubation at 4 °C on a rotator. Chromatin-antibody complexes were isolated with 20 μL of Protein-A Sepharose beads (Santa Cruz) blocked by sperm DNA and bovine serum albumin. After extensive washing, protein/DNA complexes were eluted from the beads in Elution buffer (50 mM Tris-HCl pH 8.0, 1% SDS, 10 mM EDTA) at 65 °C. Immunoprecipitated DNA was purified by phenol/chloroform extraction, and analyzed by real time quantitative PCR. The data represent mean ± STD for repeats. The primers for the ChIP-qPCR are listed below. 5′-GTGCAGAAGGATGGAACGGA-3′ (forward) and 5′-TCAAGGGCTCTCACAAACCC-3′ (reverse) for DNMT1-1; 5′-ACCAAGCTGGAGTCAAGAGC-3′ (forward) and 5′-GCTAGAAACTAGGCGGGGTG-3′ (reverse) for DNMT1-2; 5′-ACACATTGATTCCGGCCTTCT-3′ (forward) and 5′-AAGTCCAAGTCCGTTCGCAG-3′ (reverse) for UHRF1-1; 5′-ACTTTGCAAAACTTTCCCGC-3′ (forward) and 5′-ACGATTCCCGAGGGTTAGGT-3′ (reverse) for UHRF1-2.

### Total RNA Extraction and Quantitative RT-PCR

Total RNA was extracted from cells using RNAiso Plus Reagent (Takara) according to the manufacturer’s instructions. For RT–qPCR analysis, 1.5 μg total RNA were reverse-transcribed with TransScriptR One-Step gDNA Removal and cDNA Synthesis SuperMix (TransGen Biotech Co., Ltd). Gene expression levels were determined by qPCR with the CFX96 Real-Time System (Bio-Rad) using TransStart Green qPCR SuperMix (TransGen Biotech Co., Ltd) and normalized to 18 s RNA. The data represent mean ± STD for repeats. The primers for RT-PCR are listed below. 5′-GTAACCCGTTGAACCCCATT-3′ (forward) and 5′-CCATCCAATCGGTAGTAGCG-3′ (reverse) for 18 s RNA; 5′-CTGGTACGACGCGGAGAT-3′ (forward) and 5′-CGACAGTCGTTCAGAGAATCA-3′ (reverse) for human UHRF1; 5′-TGTGGCGTCTGTGAGGTG-3′ (forward) and 5′-ATGCGATTCTTGTTCTGTTTCT-3′ (reverse) for human DNMT1; 5′-CACCGGCCATACGGTGG-3′ (forward) and 5′-GGTCTTTGGAGGCGAGAGTT-3′ (reverse) for human DNMT3A; 5′- CCACACCGTGAACTCTCTGTC-3′ (forward) and 5′-GGCGCACATCATAATCGAAGA-3′ (reverse) for mouse UHRF1; 5′- CCGTGGCTACGAGGAGAAC-3′ (forward) and 5′- TTGGGTTTCCGTTTAGTGGGG-3′ (reverse) for mouse DNMT1. The primer sequences against mouse DNMT3A, and PRDM14 are described previously^35^.

### Luciferase Reporter Assay

To test if UHRF1 and DNMT1 proximal promoters were responsive to MEK inhibition and transcription factors E2F1 and SP1, the DNA fragment corresponding to −295bp to +1113 bp of the UHRF1 transcription start site (hg38, chr19:4,909,203-4,910,613) and the DNA fragment corresponding to −475bp to +1381 bp of the DNMT1 transcription start site (hg38, chr19: 10,195,554-10,193,696) were cloned into the PGL3-basic luciferase reporter. The resulting UHRF1-luc and DNMT-luc reporters were either transfected alone and treated with or without PD0325901 as in Fig. 3g and h or cotransfected with or without E2F1 or SP1 expression plasmids and treated with or without PD0325901 as in Fig. 7g and h. The luciferase assays were performed using Luciferase Assay Kit (Promega, E1501) according to the manufacturer’s instructions.

### Cell Viability Assay

Cell viability was measured by Cell Counting Kit-8 (CCK-8) (Biomike) according to manufacturer’s instructions. Cells were seeded in 96-well plates at 5 × 10^3^ cells/well and grew overnight. After treated with the inhibitors for indicted hours, 20 μL of CCK-8 solution was added and incubated for 30 min at 37 °C. Absorbance at 450 nm was measured with spectra max 190 (Molecular Devices). The data represent mean ± STD for repeats.

### Statistical analysis

All experiments were performed at least three independent times unless otherwise indicated. Statistical analysis and graphs were generated using Microsoft Excel. All statistical analyses were showed by bar graph or line graph with mean and standard deviation (STDEV.P).

## Supplementary information

Supplementary Info

## References

[1] Jones PA, Issa JP, Baylin S (2016). Targeting the cancer epigenome for therapy. Nat Rev Genet.

[2] Li E, Zhang Y (2014). DNA methylation in mammals. Cold Spring Harb Perspect Biol.

[3] Esteller M (2008). Epigenetics in cancer. N Engl J Med.

[4] Cai Y (2017). Critical threshold levels of DNA methyltransferase 1 are required to maintain DNA methylation across the genome in human cancer cells. Genome Res.

[5] Bostick M (2007). UHRF1 plays a role in maintaining DNA methylation in mammalian cells. Science.

[6] Sharif J (2007). The SRA protein Np95 mediates epigenetic inheritance by recruiting Dnmt1 to methylated DNA. Nature.

[7] Robertson KD (1999). The human DNA methyltransferases (DNMTs) 1, 3a and 3b: coordinate mRNA expression in normal tissues and overexpression in tumors. Nucleic Acids Res.

[8] Subramaniam D, Thombre R, Dhar A, Anant S (2014). DNA methyltransferases: a novel target for prevention and therapy. Front Oncol.

[9] Lin RK, Wang YC (2014). Dysregulated transcriptional and post-translational control of DNA methyltransferases in cancer. Cell Biosci.

[10] McCabe MT, Davis JN, Day ML (2005). Regulation of DNA methyltransferase 1 by the pRb/E2F1 pathway. Cancer Res.

[11] McCabe MT, Low JA, Imperiale MJ, Day ML (2006). Human polyomavirus BKV transcriptionally activates DNA methyltransferase 1 through the pRb/E2F pathway. Oncogene.

[12] Kishikawa S, Murata T, Kimura H, Shiota K, Yokoyama KK (2002). Regulation of transcription of the Dnmt1 gene by Sp1 and Sp3 zinc finger proteins. Eur J Biochem.

[13] Lin RK (2010). Dysregulation of p53/Sp1 control leads to DNA methyltransferase-1 overexpression in lung cancer. Cancer Res.

[14] Azizi M (2014). MicroRNA-148b and microRNA-152 reactivate tumor suppressor genes through suppression of DNA methyltransferase-1 gene in pancreatic cancer cell lines. Cancer Biol Ther.

[15] Chen Y (2013). Decreased miRNA-148a is associated with lymph node metastasis and poor clinical outcomes and functions as a suppressor of tumor metastasis in non-small cell lung cancer. Oncol Rep.

[16] Jeanblanc M (2005). The retinoblastoma gene and its product are targeted by ICBP90: a key mechanism in the G1/S transition during the cell cycle. Oncogene.

[17] Wang F (2012). UHRF1 promotes cell growth and metastasis through repression ofp16(ink(4)a) in colorectal cancer. Ann Surg Oncol.

[18] Achour M (2008). The interaction of the SRA domain of ICBP90 with a novel domain of DNMT1 is involved in the regulation of VEGF gene expression. Oncogene.

[19] Jin W (2010). UHRF1 is associated with epigenetic silencing of BRCA1 in sporadic breast cancer. Breast Cancer Res Treat.

[20] Sabatino L (2012). UHRF1 coordinates peroxisome proliferator activated receptor gamma (PPARG) epigenetic silencing and mediates colorectal cancer progression. Oncogene.

[21] Zhang Y (2014). Upregulated UHRF1 promotes bladder cancer cell invasion by epigenetic silencing of KiSS1. PLoS One.

[22] Ashraf W (2017). The epigenetic integrator UHRF1: on the road to become a universal biomarker for cancer. Oncotarget.

[23] Mousli M (2003). ICBP90 belongs to a new family of proteins with an expression that is deregulated in cancer cells. Br J Cancer.

[24] Unoki M, Nishidate T, Nakamura Y (2004). ICBP90, an E2F-1 target, recruits HDAC1 and binds to methyl-CpG through its SRA domain. Oncogene.

[25] Park, S. A. *et al*. E2F8 as a Novel Therapeutic Target for Lung Cancer. *J Natl Cancer Inst* **107**, 10.1093/jnci/djv151 (2015). 10.1093/jnci/djv151 PMC4651101 26089541

[26] Wu SM (2015). Negative modulation of the epigenetic regulator, UHRF1, by thyroid hormone receptors suppresses liver cancer cell growth. Int J Cancer.

[27] Sanders DA (2015). FOXM1 binds directly to non-consensus sequences in the human genome. Genome Biol.

[28] Deng W (2015). Quantitative proteomic analysis of the metastasis-inhibitory mechanism of miR-193a-3p in non-small cell lung cancer. Cell Physiol Biochem.

[29] Goto Y (2016). The microRNA signature of patients with sunitinib failure: regulation of UHRF1 pathways by microRNA-101 in renal cell carcinoma. Oncotarget.

[30] Matsushita R (2016). Regulation of UHRF1 by dual-strand tumor-suppressor microRNA-145 (miR-145-5p and miR-145-3p): Inhibition of bladder cancer cell aggressiveness. Oncotarget.

[31] Wang X (2015). MiR-124 exerts tumor suppressive functions on the cell proliferation, motility and angiogenesis of bladder cancer by fine-tuning UHRF1. FEBS J.

[32] Zhou L (2013). Regulation of UHRF1 by miR-146a/b modulates gastric cancer invasion and metastasis. FASEB J.

[33] Zhu M, Xu Y, Ge M, Gui Z, Yan F (2015). Regulation of UHRF1 by microRNA-9 modulates colorectal cancer cell proliferation and apoptosis. Cancer Sci.

[34] Ying QL (2008). The ground state of embryonic stem cell self-renewal. Nature.

[35] Ficz G (2013). FGF signaling inhibition in ESCs drives rapid genome-wide demethylation to the epigenetic ground state of pluripotency. Cell Stem Cell.

[36] Habibi E (2013). Whole-genome bisulfite sequencing of two distinct interconvertible DNA methylomes of mouse embryonic stem cells. Cell Stem Cell.

[37] Hackett JA (2013). Synergistic mechanisms of DNA demethylation during transition to ground-state pluripotency. Stem Cell Reports.

[38] Leitch HG (2013). Naive pluripotency is associated with global DNA hypomethylation. Nat Struct Mol Biol.

[39] von Meyenn F (2016). Impairment of DNA Methylation Maintenance Is the Main Cause of Global Demethylation in Naive Embryonic Stem Cells. Mol Cell.

[40] Graf U (2017). Pramel7 mediates ground-state pluripotency through proteasomal-epigenetic combined pathways. Nat Cell Biol.

[41] Bos J (1989). L. ras oncogenes in human cancer: a review. Cancer Res.

[42] Davies H (2002). Mutations of the BRAF gene in human cancer. Nature.

[43] Steelman LS (2004). JAK/STAT, Raf/MEK/ERK, PI3K/Akt and BCR-ABL in cell cycle progression and leukemogenesis. Leukemia.

[44] Woodgett JR (1990). Molecular cloning and expression of glycogen synthase kinase-3/factor A. EMBO J.

[45] Embi N, Rylatt DB, Cohen P (1980). Glycogen synthase kinase-3 from rabbit skeletal muscle. Separation from cyclic-AMP-dependent protein kinase and phosphorylase kinase. Eur J Biochem.

[46] McCubrey JA (2014). GSK-3 as potential target for therapeutic intervention in cancer. Oncotarget.

[47] Yamaji M (2013). PRDM14 ensures naive pluripotency through dual regulation of signaling and epigenetic pathways in mouse embryonic stem cells. Cell Stem Cell.

[48] Morris EJ (2013). Discovery of a novel ERK inhibitor with activity in models of acquired resistance to BRAF and MEK inhibitors. Cancer Discov.

[49] Okamura Y (2015). COXPRESdb in 2015: coexpression database for animal species by DNA-microarray and RNAseq-based expression data with multiple quality assessment systems. Nucleic Acids Res.

[50] Zhu, Q. *et al*. Targeted exploration and analysis of large cross-platform human transcriptomic compendia. *Nat Methods* **12**, 211–214, 213 p following 214, 10.1038/nmeth.3249 (2015). 10.1038/nmeth.3249 PMC4768301 25581801

[51] Tanaka T (1996). The extracellular signal-regulated kinase pathway phosphorylates AML1, an acute myeloid leukemia gene product, and potentially regulates its transactivation ability. Mol Cell Biol.

[52] Wang S, Nath N, Minden A, Chellappan S (1999). Regulation of Rb and E2F by signal transduction cascades: divergent effects of JNK1 and p38 kinases. EMBO J.

[53] Merchant JL, Du M, Todisco A (1999). Sp1 phosphorylation by Erk 2 stimulates DNA binding. Biochem Biophys Res Commun.

[54] Chung J, Uchida E, Grammer TC, Blenis J (1997). STAT3 serine phosphorylation by ERK-dependent and -independent pathways negatively modulates its tyrosine phosphorylation. Mol Cell Biol.

[55] Zheng H, Chu J, Zeng Y, Loh HH, Law PY (2010). Yin Yang 1 phosphorylation contributes to the differential effects of mu-opioid receptor agonists on microRNA-190 expression. J Biol Chem.

[56] Stoeckius M (2012). Essential roles of Raf/extracellular signal-regulated kinase/mitogen-activated protein kinase pathway, YY1, and Ca2+ influx in growth arrest of human vascular smooth muscle cells by bilirubin. J Biol Chem.

[57] Yoon S, Seger R (2006). The extracellular signal-regulated kinase: multiple substrates regulate diverse cellular functions. Growth Factors.

[58] Yang SH, Sharrocks AD, Whitmarsh AJ (2003). Transcriptional regulation by the MAP kinase signaling cascades. Gene.

[59] Yang SH, Sharrocks AD, Whitmarsh AJ (2013). MAP kinase signalling cascades and transcriptional regulation. Gene.

[60] Fernandez-Medarde A, Santos E (2011). Ras in cancer and developmental diseases. Genes Cancer.

[61] Prior IA, Lewis PD, Mattos C (2012). A comprehensive survey of Ras mutations in cancer. Cancer Res.

[62] Lu R (2007). Inhibition of the extracellular signal-regulated kinase/mitogen-activated protein kinase pathway decreases DNA methylation in colon cancer cells. J Biol Chem.

