# Supplementary material for: Activated MEK/ERK Pathway Drives Widespread and Coordinated Overexpression of UHRF1 and DNMT1 in Cancer cells
Source: Sci Rep. 2019 Jan 29;9:907. doi: 10.1038/s41598-018-37258-3 (PMC6351616; doi:10.1038/s41598-018-37258-3)
Supplement: Supplementary file 1 — Supplementary Info [file 41598_2018_37258_MOESM1_ESM.pdf]

Supplementary File for

Activated MEK/ERK Pathway Drives Widespread and Coordinated Overexpression of UHRF1 and DNMT1 in Cancer cells

Jialun Li, Ruiping Wang, Xueli Hu, Yingying Gao, Zhen Wang, Jiwen Li, Jiemin Wong

Contains supplementary Figures S1-S8.

(a)

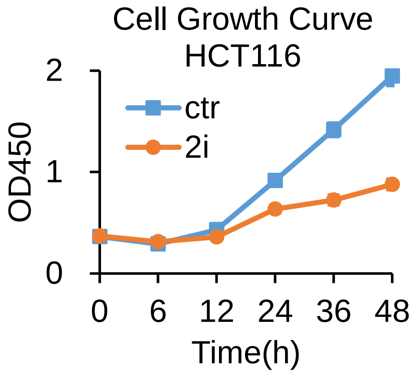

(b)

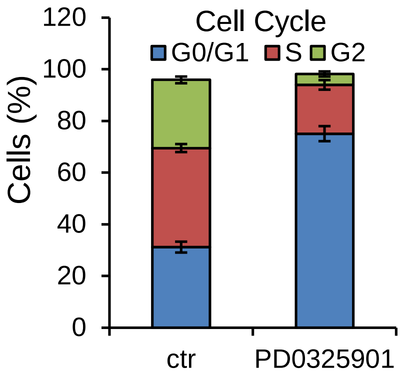

Supplementary Figure S1. 2i treatment inhibits HCT116 proliferation. (**a**) HCT116 cell were treated with or without 2i for the indicated time and the relative level of viable cells was measured by Cell Counting Kit-8 (CCK-8). (**b**) HCT116 cells were treated with PD0325901 for 12 hours before harvested for analysis of the cell cycle by FACS.

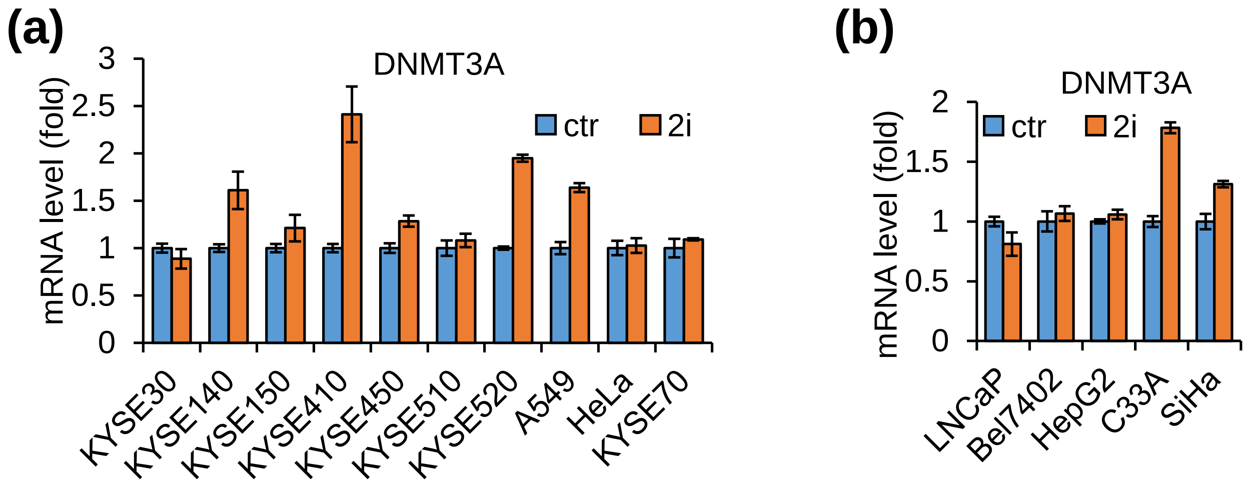

Supplementary Figure S2. The effect of 2i treatment on DNMT3A transcription in various cancer cells. All the cells were treated with or without 2i for 2 days before harvested for RT-qPCR analysis.

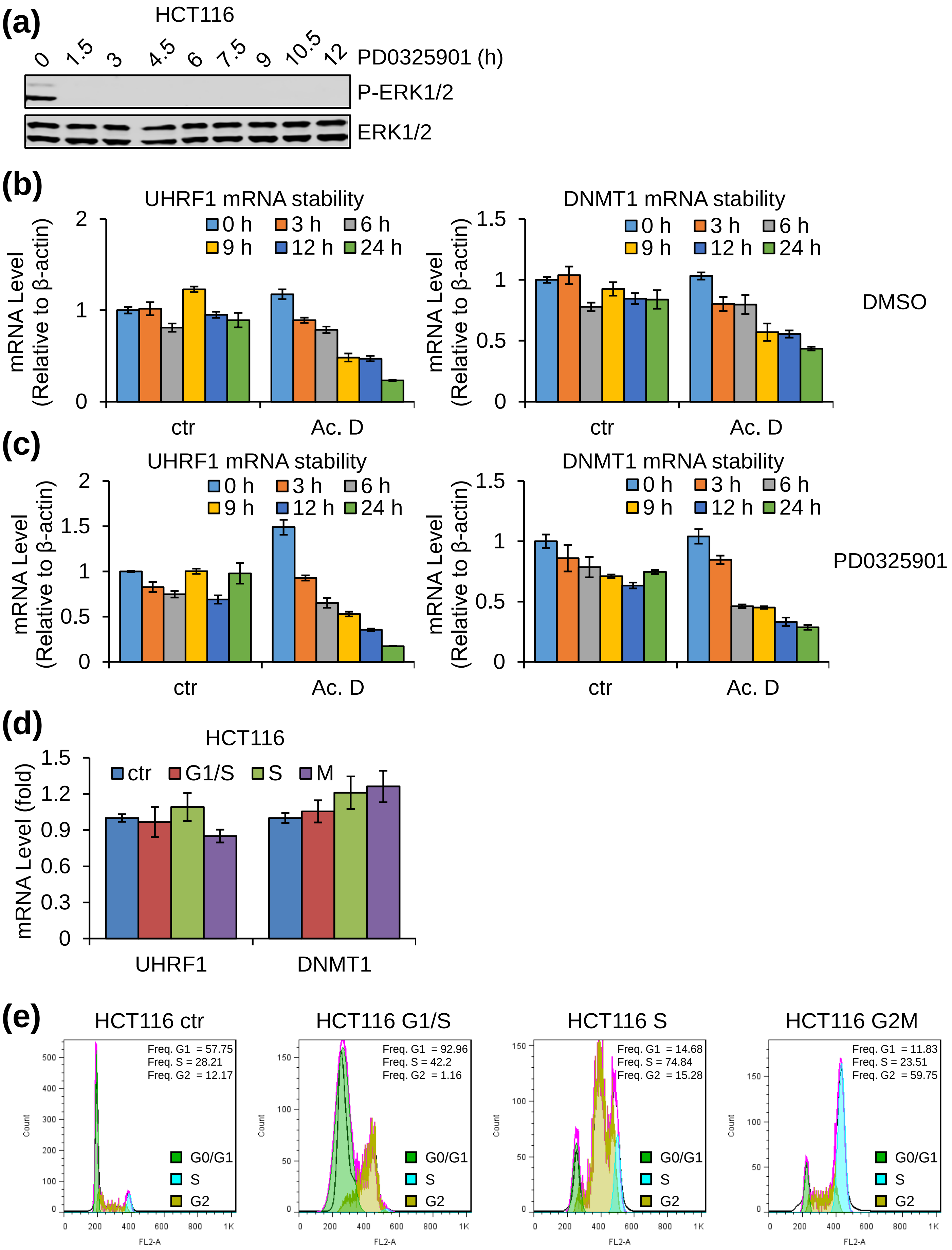
(a)
HCT116
0 1.5 3 4.5 6 7.5 9 10.5 12 PD0325901 (h)
P-ERK1/2
ERK1/2
(b)
UHRF1 mRNA stability
DNMT1 mRNA stability
0 h 3 h 6 h 9 h 12 h 24 h
mRNA Level (Relative to β-actin)
ctr
Ac. D
DMSO
(c)
UHRF1 mRNA stability
DNMT1 mRNA stability
PD0325901
(d)
HCT116
ctr G1/S S M
mRNA Level (fold)
UHRF1
DNMT1
(e)
HCT116 ctr
Freq. G1 = 57.75
Freq. S = 28.21
Freq. G2 = 12.17
HCT116 G1/S
Freq. G1 = 92.96
Freq. S = 42.2
Freq. G2 = 1.16
HCT116 S
Freq. G1 = 14.68
Freq. S = 74.84
Freq. G2 = 15.28
HCT116 G2M
Freq. G1 = 11.83
Freq. S = 23.51
Freq. G2 = 59.75
G0/G1
S
G2
Count
FL2-A

Supplementary Figure S3. The effect of MEK inhibitor PD0325901 on UHRF1 and DNMT1 mRNA stability and the effect of cell cycle on UHRF1 and DNMT1 expression. (**a**) Effective and rapid inhibition of ERK activity by PD0325901. HCT116 cells were treated with PD0325901 for the indicated time before harvested for western blotting analysis. (**b**,**c**) Treatment with PD0325901 had no effect on UHRF1 and DNMT1 mRNA stability. HCT116 cells were treated with DMSO and actinomycin D (Ac.D) in the absence or presence of PD0325901 for various times as indicated and subjected for RT-qPCR analysis. (**d**,**e**) The levels of UHRF1 and DNMT1 mRNAs were not significantly changed with cell cycle. HCT116 cells were synchronized to G1/S boundary by aphidicolin (1 μg/mL) treatment for 18 hours and then released into S phase by culturing in fresh medium for 4 hours. For G2/M phase enrichment, the cells were treated with Nocodazole (50 ng/mL) for 18 hours. Finally, the cells were subjected for analysis of mRNA level by RT-qPCR (**d**) and cell cycle by FACS (**e**).

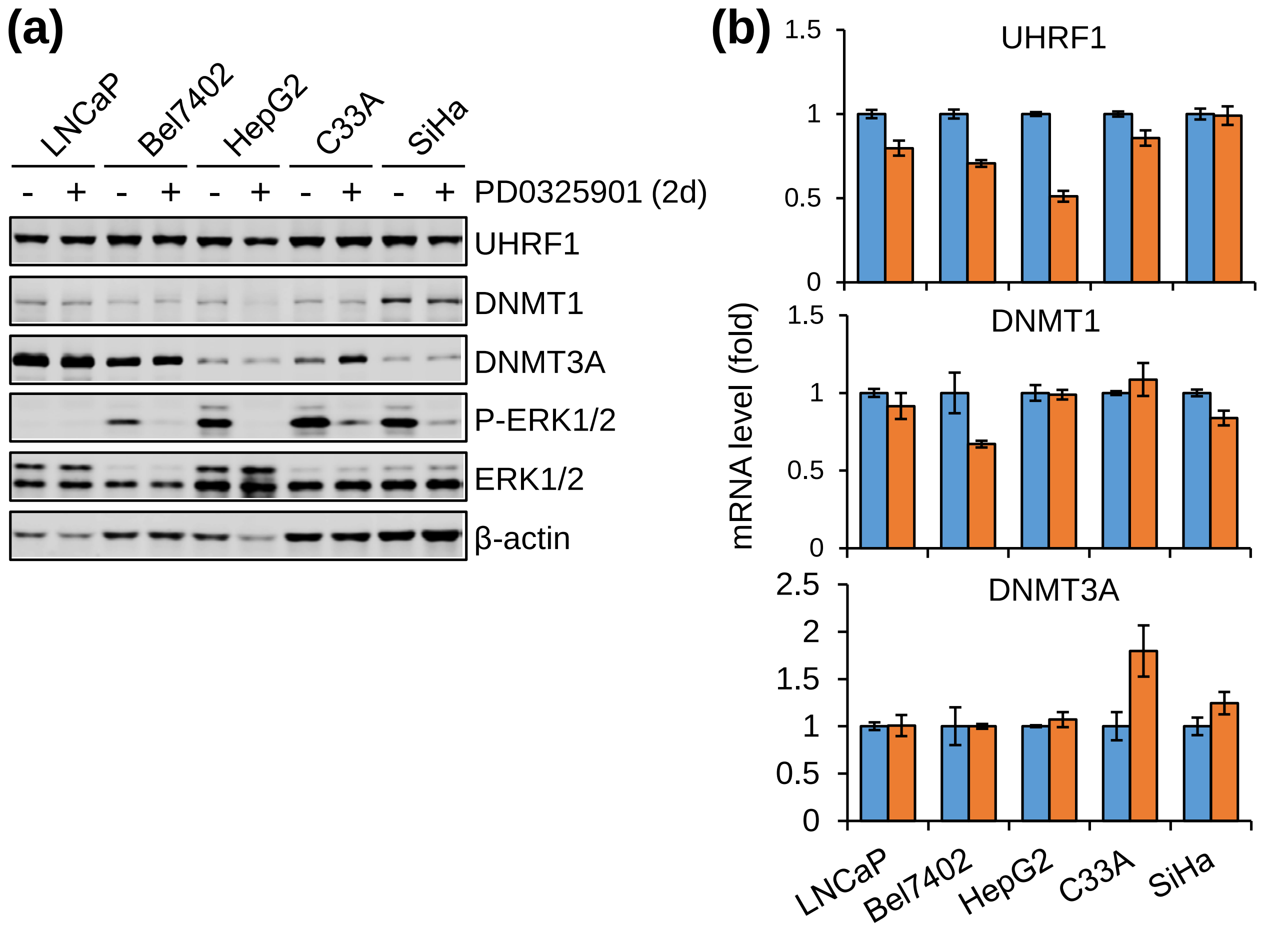

Supplementary Figure S4. A subset of cell lines are insensitive to PD0325901-induced reduction of UHRF1 and DNMT1 proteins. (**a**,**b**) LNCaP, BEL7402, HepG2, C33A and SiHa cells were treated with PD0325901 for 2 days before harvested for analysis of the levels of various proteins by western blotting using antibodies as indicated (**a**) and the levels of mRNAs by qRT-PCR (**b**). Note that PD0325901 inhibited ERK1/2 phosphorylation in all but LNCaP cells.

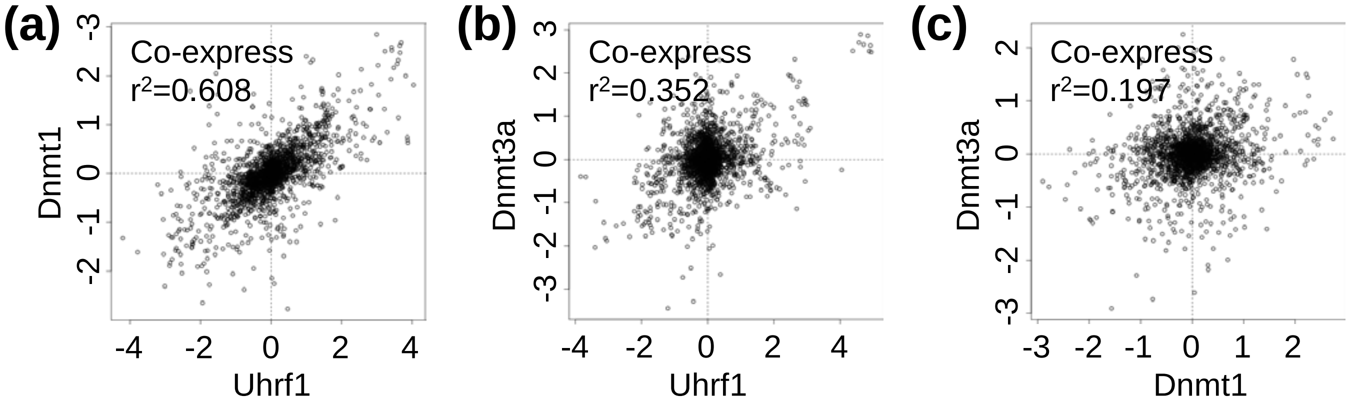

Supplementary Figure S5. Co-expression analysis of Uhrf1, Dnmt1 and Dnmt3a in mouse tissues. (**a**-**c**)The COXPRESdb was used for analyzing the co-expression status between mouse Uhrf1 and Ddnmt1 (**a**), Dnmt3a and Uhrf1 (**b**) and Dnmt3a and Dnmt1 (**c**) in Mus musculus.

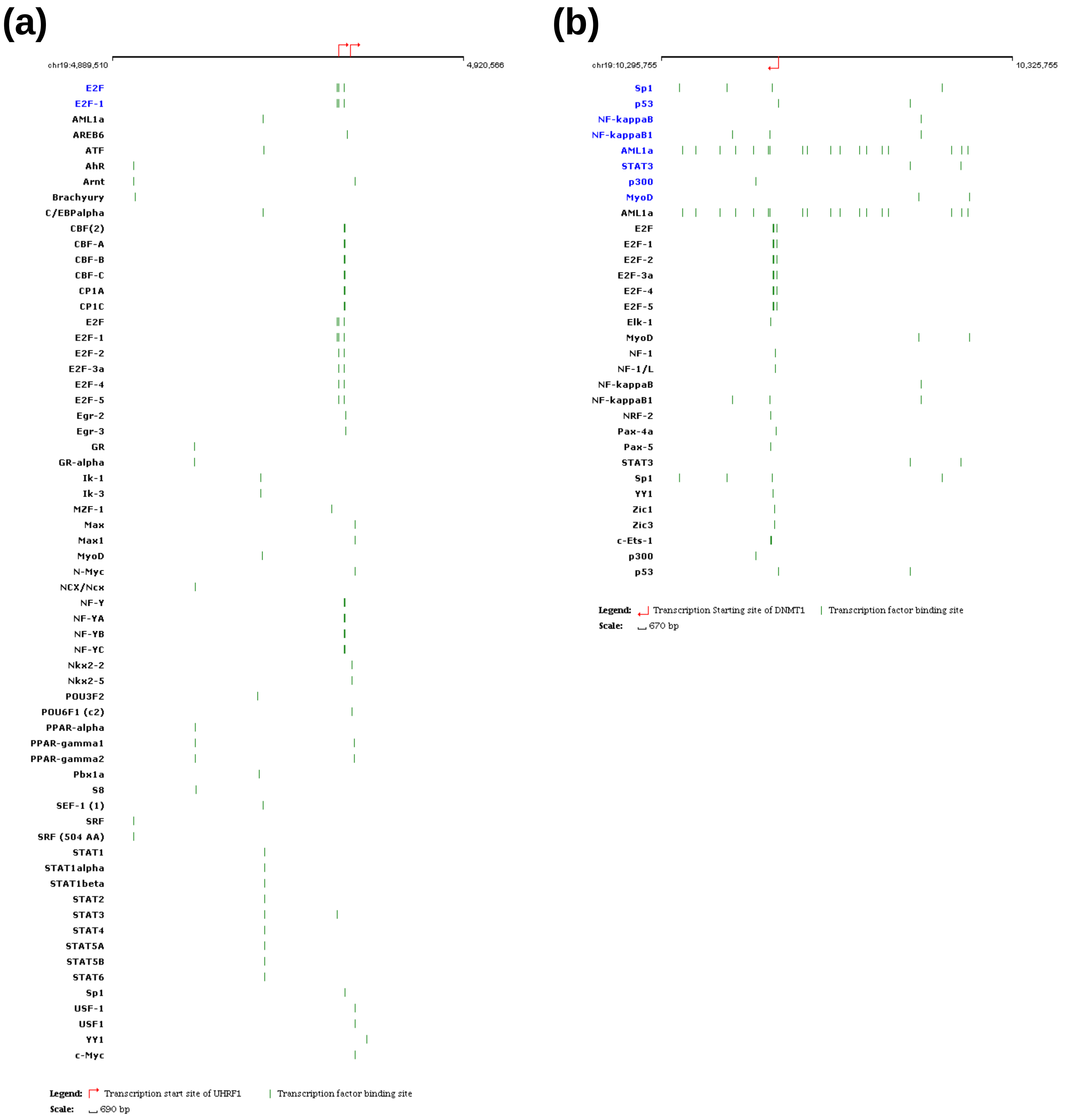

Supplementary Figure S6. The list of common putative transcription factor-binding sites within +5 kb upstream and -3kb downstream of the transcription start sites of the UHRF1 (**a**) and DNMT1 (**b**) genes. The analysis was performed using SABiosciences proprietary database (http://www.sabiosciences.com/chipqpcrsearch.php).

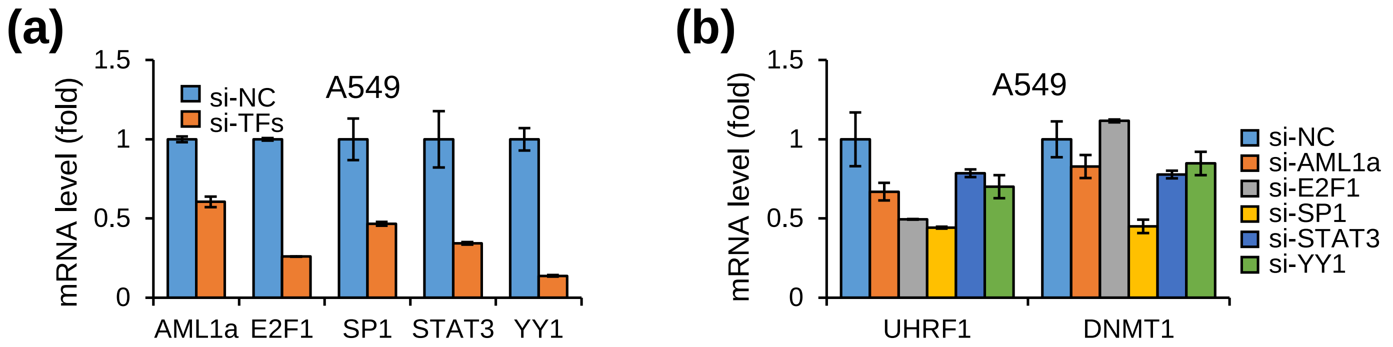

Supplementary Figure S7. Multiple MEK/ERK downstream transcription factors are involved in transcriptional activation of UHRF1 and DNMT1 in A549 cells. (**a**) qRT-PCR analysis showing efficient knockdown of corresponding transcription factors in A549 cells by specific siRNA against AML1a, E2F1, SP1, STAT3 and YY1, respectively. (**b**) qRT-PCR analysis showing the effect on UHRF1 and DNMT1 expression in A549 cells upon knockdown of each of the transcription factors.

Supplementary Figure S8. Uncropped blots used for this study.

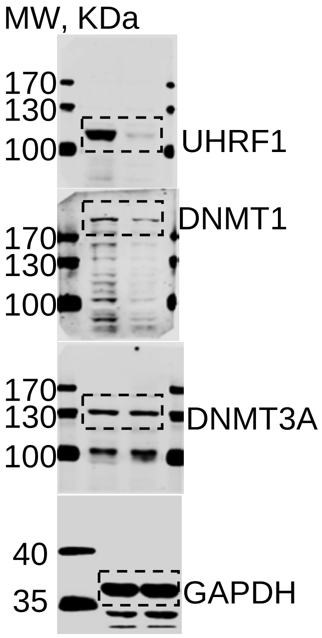

For Figure 1a.

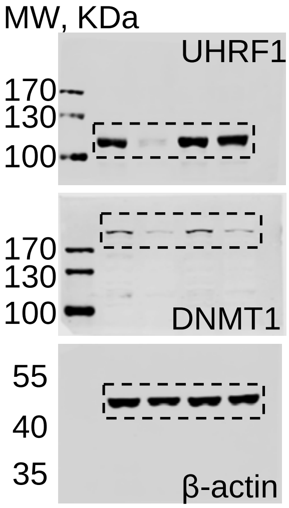

For Figure 1b.

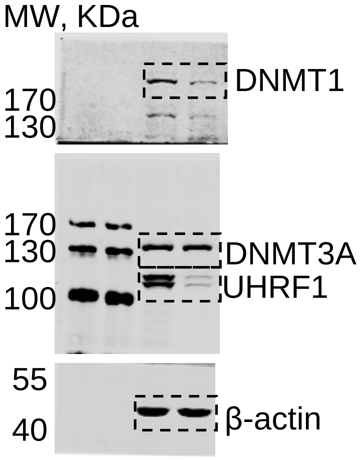

For Figure 1d.

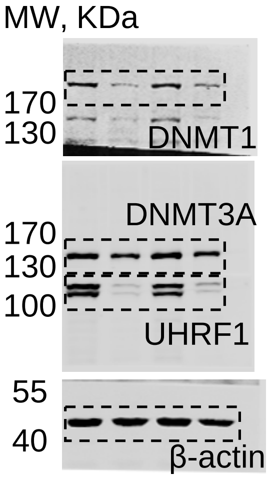

For Figure 1e.

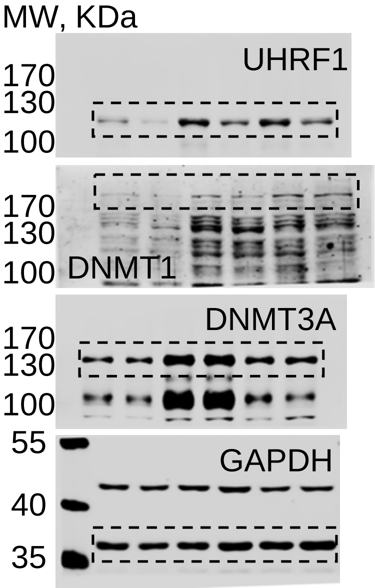

For Figure 2a.

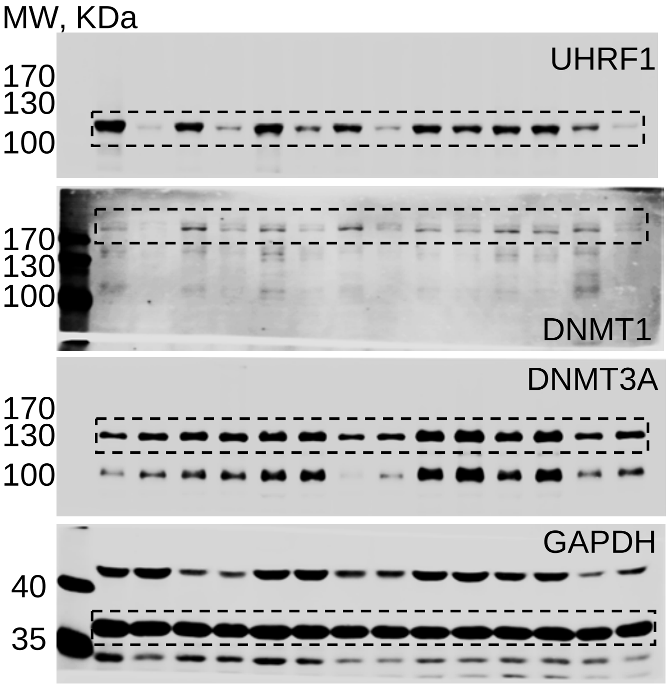

For Figure 2b.

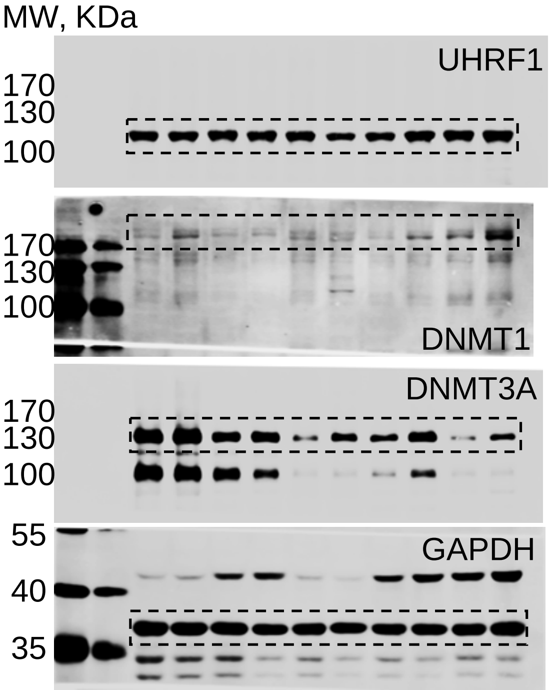

For Figure 2e.

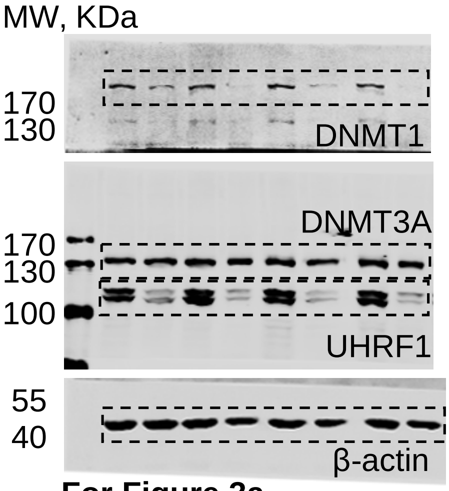

For Figure 3a.

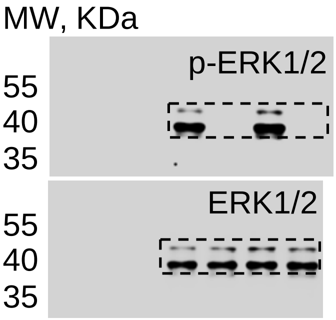

For Figure 3c.

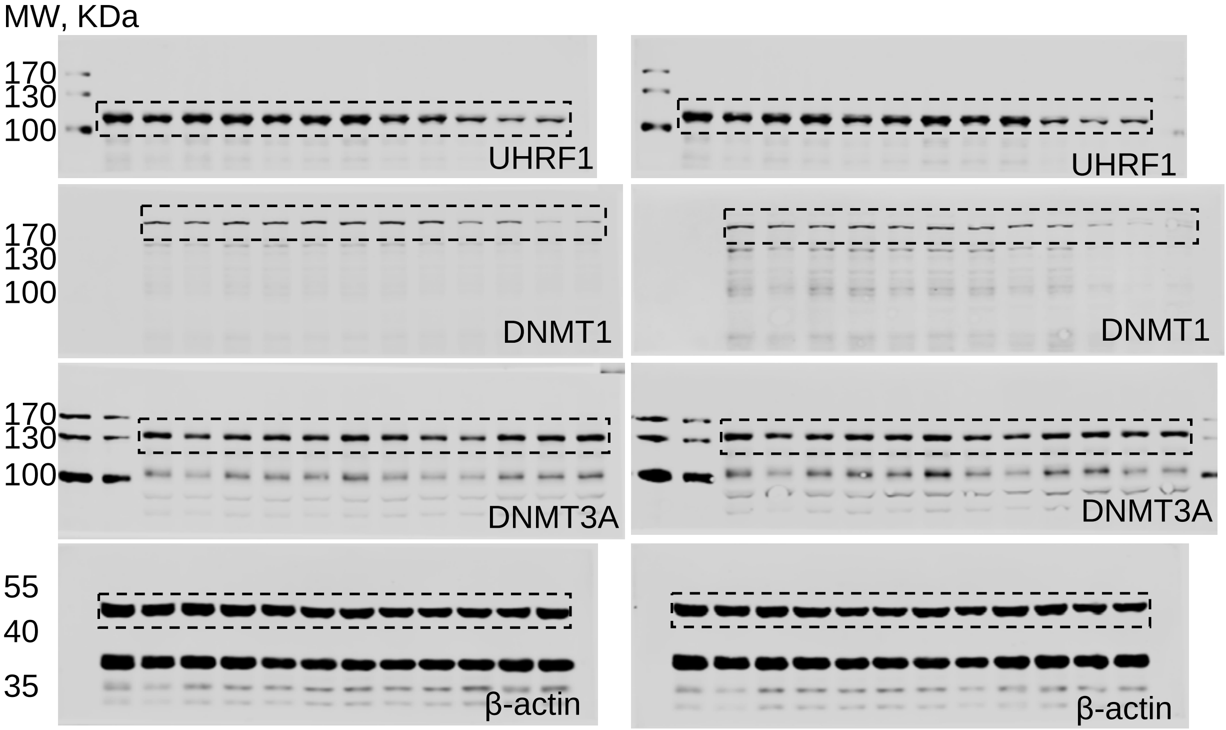

For Figure 3d.

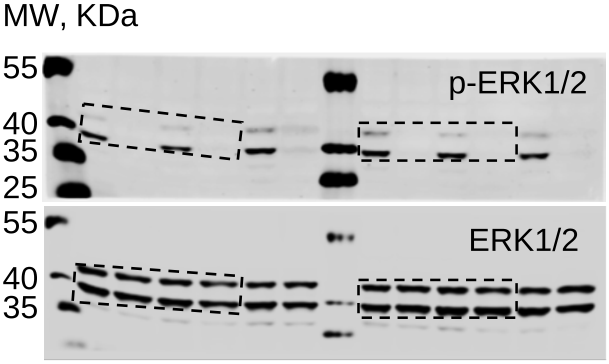

For Figure 3g. **For Figure 3h.**

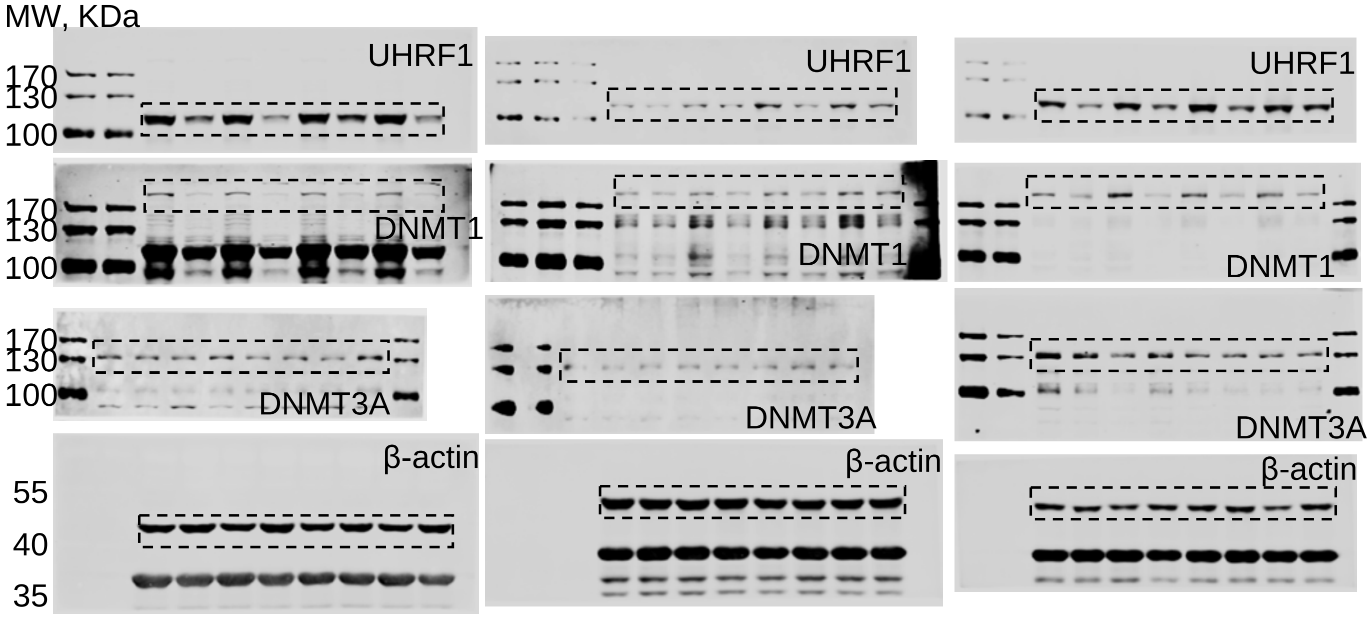

For Figure 4a.

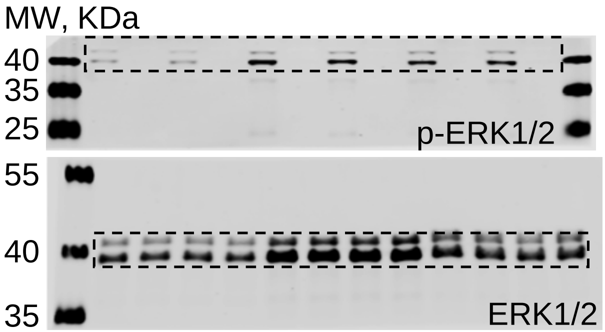

For Figure 4c.

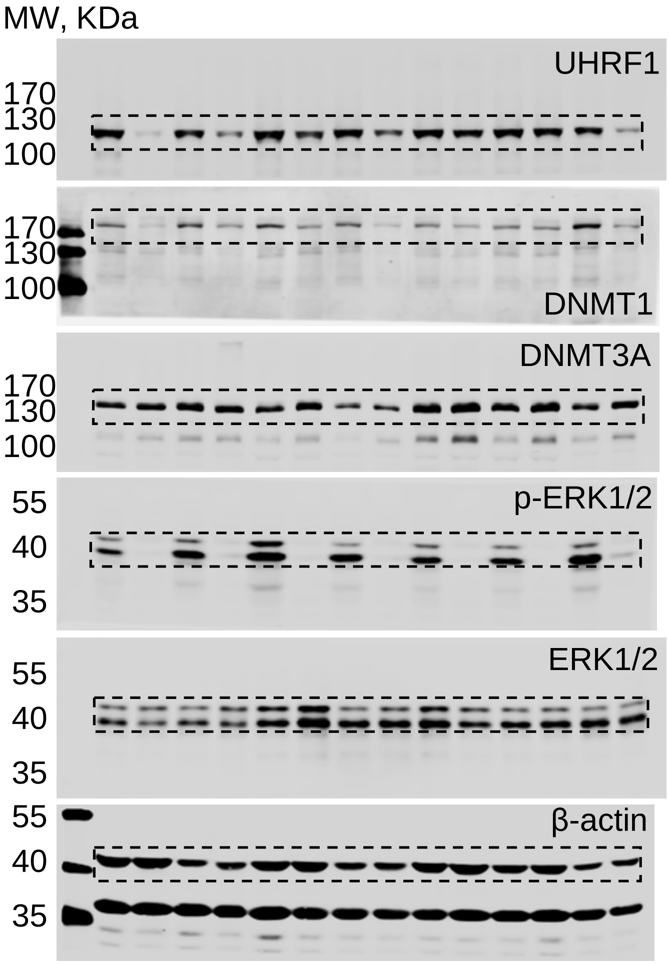

For Figure 4d.

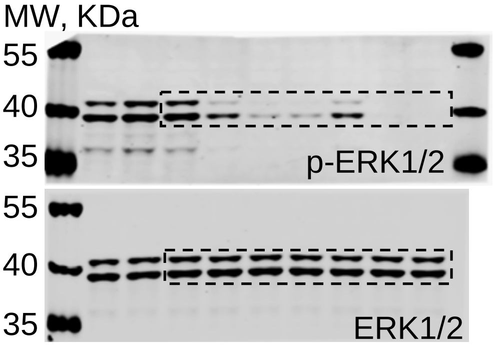

For Figure 5a.

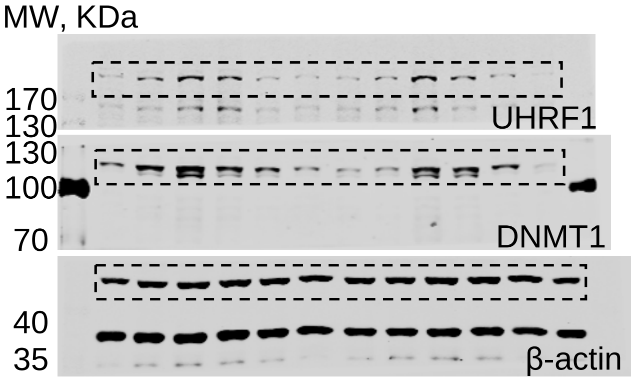

For Figure 5c.

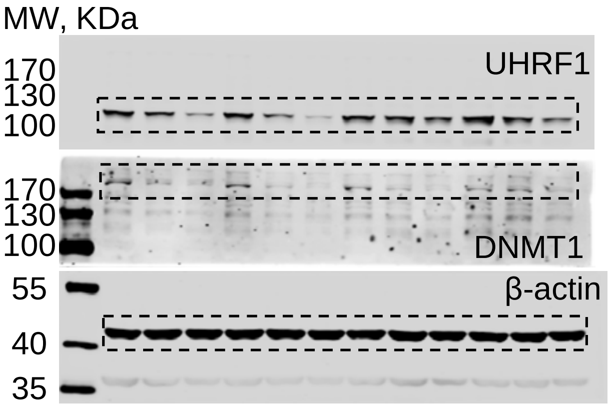

For Figure 5d.

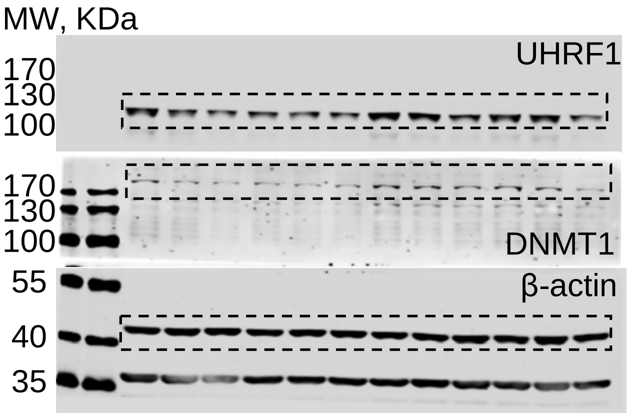

For Figure 5f.

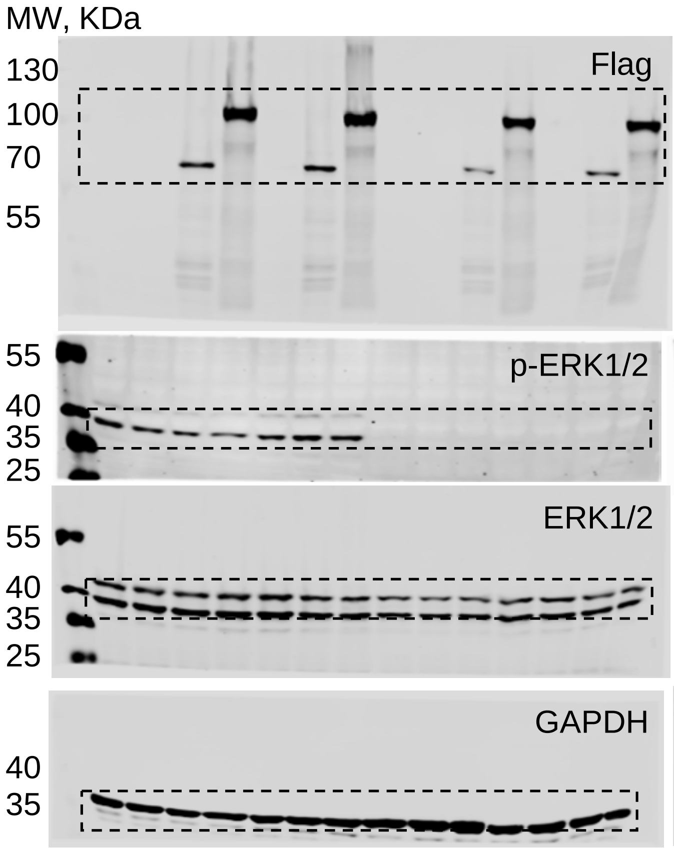

For Figure 7g.

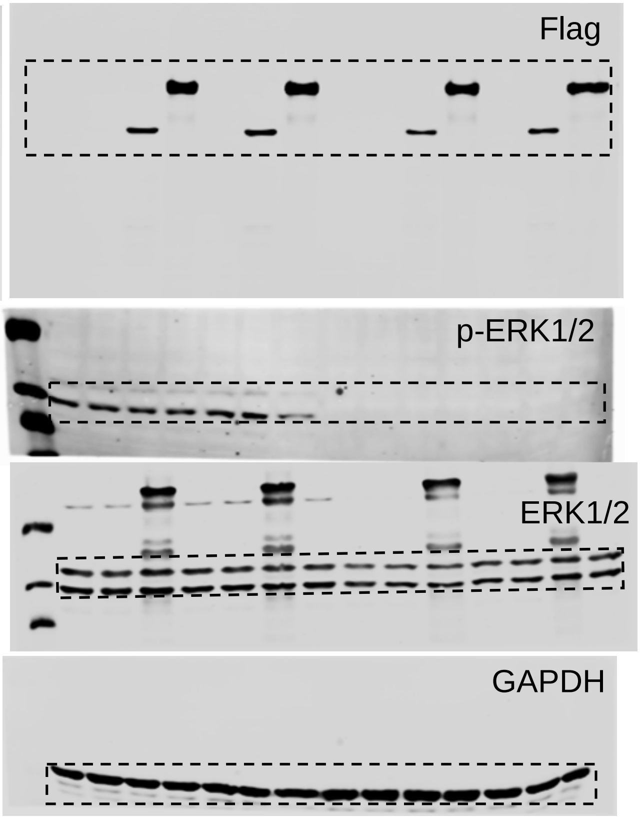

For Figure 7h.

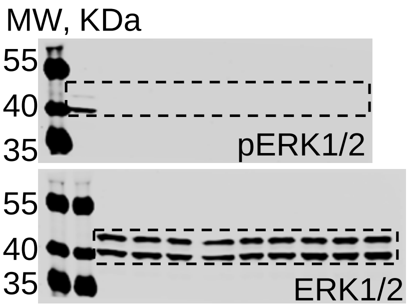

For Figure S3a.

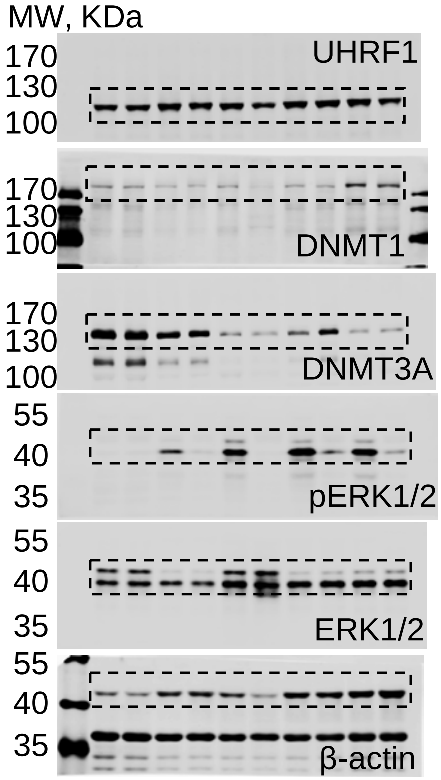

For Figure S4a.
